# Hypoxia-responsive nanoreactors based on self-enhanced photodynamic sensitization and triggered ferroptosis for cancer synergistic therapy

**DOI:** 10.1186/s12951-021-00952-y

**Published:** 2021-07-08

**Authors:** Xiaoyan Wang, Ming Wu, Xiaolong Zhang, Feida Li, Yongyi Zeng, Xinyi Lin, Xiaolong Liu, Jingfeng Liu

**Affiliations:** 1 School of Life Sciences, Fujian Agriculture and Forestry University, Fuzhou, 350002 People’s Republic of China; 2 The United Innovation of Mengchao Hepatobiliary Technology Key Laboratory of Fujian Province, Mengchao Hepatobiliary Hospital of Fujian Medical University, Fuzhou, 350025 People’s Republic of China; 3 Mengchao Med-X Center, Fuzhou University, Fuzhou, 350116 People’s Republic of China; 4 Fujian Institute of Research on the Structure of Matter, Chinese Academy of Sciences, Fuzhou, 350002 China; 5 Fujian Cancer Hospital & Fujian Medical University Cancer Hospital, Fuzhou, 350014 People’s Republic of China

**Keywords:** Oxidation treatment, Photodynamic therapy (PDT), Ferroptosis, Hypoxia-responsive, Protein-based nanoparticle

## Abstract

**Background:**

Photodynamic therapy (PDT), a typical reactive oxygen species (ROS)-dependent treatment with high controllability, has emerged as an alternative cancer therapy modality but its therapeutic efficacy is still unsatisfactory due to the limited light penetration and constant oxygen consumption. With the development of another ROS-dependent paradigm ferroptosis, several efforts have been made to conquer the poor efficacy by combining these two approaches; however the biocompatibility, tumor-targeting capacity and clinical translation prospect of current studies still exist great concerns. Herein, a novel hypoxia-responsive nanoreactor BCFe@SRF with sorafenib (SRF) loaded inside, constructed by covalently connecting chlorin e6 conjugated bovine serum albumin (BSA-Ce6) and ferritin through azobenzene (Azo) linker, were prepared to offer unmatched opportunities for high-efficient PDT and ferroptosis synergistic therapy.

**Results:**

The designed BCFe@SRF exhibited appropriate size distribution, stable dispersity, excellent ROS generation property, controllable drug release capacity, tumor accumulation ability, and outstanding biocompatibility. Importantly, the BCFe@SRF could be degraded under hypoxia environment to release BSA-Ce6 for laser-triggered PDT, ferritin for iron-catalyzed Fenton reaction and SRF for tumor antioxidative defense disruption. Meanwhile, besides PDT effects, it was found that BCFe@SRF mediated treatment upon laser irradiation in hypoxic environment not only could accelerate lipid peroxidation (LPO) generation but also could deplete intracellular glutathione (GSH) and decrease glutathione peroxidase (GPX4) expression, which was believed as three symbolic events during ferroptosis. All in all, the BCFe@SRF nanoreactor, employing multiple cascaded pathways to promote intracellular ROS accumulation, presented remarkably outstanding antitumor effects both in vitro and in vivo.

**Conclusion:**

BCFe@SRF could serve as a promising candidate for synergistic PDT and ferroptosis therapy, which is applicable to boost oxidative damage within tumor site and will be informative to future design of ROS-dependent therapeutic nanoplatforms.

**Graphic abstract:**

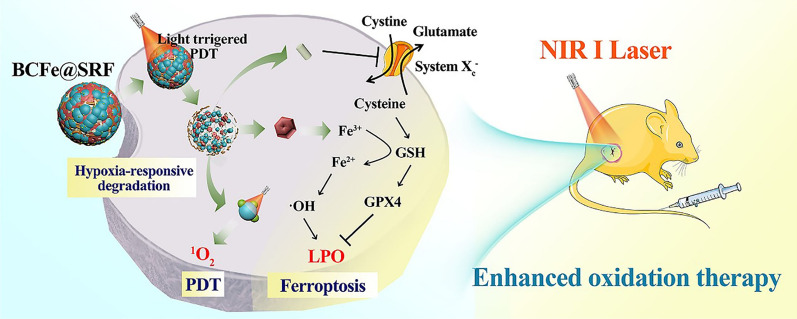

**Supplementary Information:**

The online version contains supplementary material available at 10.1186/s12951-021-00952-y.

## Background

Reactive oxygen species (ROS)-dependent treatment, as an alternative cancer therapeutic paradigm, has recently gained tremendous attentions [1, 2]. ROS is a family of incomplete reductive molecular forms of oxygen including singlet oxygen (^1^O_2_), hydroxyl radical (⋅OH), superoxide anion (O_2_^⋅−^), hydrogen peroxide (H_2_O_2_), etc., which cause oxidative damage to cells through destroying lipid, protein and DNA structures [1, 3, 4]. Photodynamic therapy (PDT), a typical oxidative therapeutic modality, employs photo-activated photosensitizers (PSs) to transfer the absorbing laser energy to surrounding oxygen molecules and subsequently generate high levels of ^1^O_2_ to kill cancer cells [5, 6]. As is well known, PDT can be operated within a controllably restricted area so that it presents enhanced selectivity and limited side effects [7, 8]. However, the low tumor accumulation of PSs after intravenous injection, limited light penetration and the constant oxygen consumption during PDT make it difficult to produce enough ROS in vivo [7–9]. In addition, cancer cells have oxidation defense systems, expressing high concentration of intracellular antioxidant glutathione (GSH), which would hamper the performance of ROS and significantly compromise PDT efficacy [10, 11]. Conceivably, boosting ROS production within tumor sites and simultaneously disrupting the oxidation defense of tumor cells should be a promising strategy to improve the antitumor efficiency.

Chemodynamic therapy (CDT), an emerging oxidation therapy depending on the so-called Fenton-type reaction, utilizes transition metals (e.g. Fe, Mn, Cu, Ni, Co) as catalysts to convert the mild oxidant H_2_O_2_ into highly toxic ·OH to kill cancer cells [12–19]. CDT generates ROS originating from H_2_O_2_ which is highly abundant in the tumor microenvironment (TME) instead of oxygen, so that it is not limited by hypoxia with tumor-site-specific ROS generation property [20]. Therefore, CDT has enormous potential to combine with PDT to amplify the oxidative stress within tumor. As reported, Fe is the most suitable Fenton-type reaction catalyst for therapeutic purpose [21], which could produce ·OH to induce lipid peroxidation (LPO) in tumor under high level of H_2_O_2_. Different from apoptotic cell death which is believed as the major mechanism of PDT [11], this iron-dependent LPO-associated oxidative damage causes a non-apoptotic form of regulated cell death type that is newly defined as ferroptosis [21–25]. Recently discovered ferroptosis-inducing agents mainly include iron-based materials (e.g. inorganic iron-involved nanoparticles (NPs), Fe-metal–organic frameworks (Fe-MOFs), iron storage or transport proteins) [1, 3, 20, 24–26] and certain small molecule drugs (e.g. sorafenib (SRF), erastin, sulfasalazine, artemisinin) [23, 27–29], the former accelerating iron-catalyzed ROS production and the latter inhibiting GSH biosynthesis and then decreasing glutathione peroxidase 4 (GPX4, the lipid repair enzyme) expression, eventually leading to large amounts of intracellular LPO accumulation. Owing to the homologous ROS production modality with tumor specificity, integrating PDT and ferroptosis therapy may provide a huge opportunity to amplify oxidative stress to more thoroughly eliminate tumor cells.

Nanoparticle (NP) platforms offer an unmatched potential for tumor-targeting simultaneous delivery of PS, exogenous iron and small GSH/GPX4 reduction molecule [30–32]. Recently, several Fe-MOFs or Fe-based inorganic NPs have been emerged for PDT and ferroptosis combining therapy, achieving significantly enhanced therapeutic outcomes in tumor oxidation treatments [11, 20, 26–29], however, the biocompatibility, tumor-targeting capacity and clinical translation prospect of which still exist great concerns. Additionally, these nanosystems utilized the acid condition or high concentration of matrix metalloproteinase (MMP) of TME to trigger cargo release, but these differentiations between normal tissues and tumor tissues are not significant enough and easily be affected by the tumor heterogeneity [33–35]. Therefore, novel strategies based on more biocompatible nanoplatforms with more specific responsiveness property are needed for synergistic therapy.

Protein-based nanocarriers have rapidly appeared as charming drug delivery vehicles due to their high structural stability, excellent biocompatibility, fantastic biodegradability and easy tailorability [36–39]. Ferritin, an iron storage and transport protein, which could be specifically recognized by transferrin receptor 1 (TfR1) overexpressed cancer cells, has been investigated as a novel type of tumor-targeting platform [40, 41]. In addition, ferritin can release stored iron to induce ferroptosis by intracellular autophagic degradation [42–45]. Therefore, the intrinsic natural iron storage and tumor specific recognition features of ferritin make it more outstanding than inorganic or MOF materials in ferroptosis treatment [46]. Meanwhile, SRF, a multikinase inhibitor, is usually used as the first-line drug in clinic for hepatocellular carcinoma but still suffers from drug resistances and serious adverse side effects, leading to unsatisfactory clinical outcomes [47–49]. Recent studies have revealed that SRF could indirectly increase LPO accumulation for ferroptosis by decreasing GSH/GPX4 [23, 28, 29], which could be combined with PDT to further boost ROS accumulation and conquer the poor efficacy of mono-therapy. On the other hand, the significantly anabatic oxygen consumption during PDT can be harnessed by hypoxia-responsive drug vehicles to achieve on-demand drug release, avoiding the insufficient difference between normal tissues and tumor tissues [50–54].

**Fig. 1:**
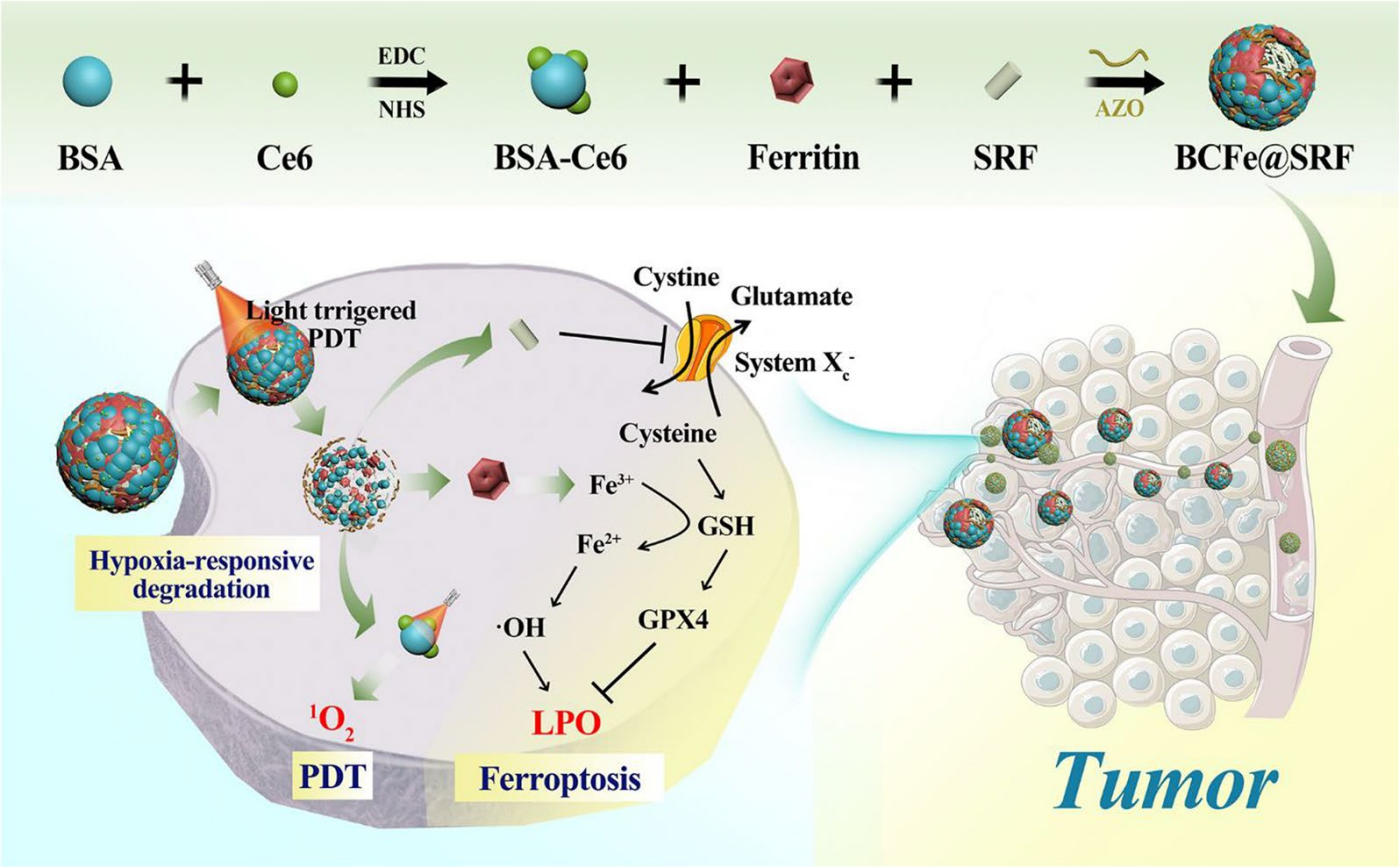
Fabrication procedures and antitumor mechanisms of BCFe@SRF nanoreactor for the designed synergistic PDT and ferroptosis therapy

Herein, a novel protein-based nanoreactor BCFe@SRF was developed by covalently crosslinking chlorin e6 (Ce6, a typical PS) [8] conjugated bovine serum albumin (BSA) and ferritin by hypoxia-responsive unit azobenzene (Azo), together with the SRF encapsulated inside the protein shell (Fig. 1). The designed BCFe@SRF exhibited outstanding biocompatibility and excellent tumor targeting efficiency. Under hypoxic environment, especially in oxygen-consumed PDT process, the BCFe@SRF could be degraded to expose more Ce6 for laser-triggered PDT and simultaneously release ferritin for iron-catalyzed Fenton reaction, producing more ROS in tumor. Additionally, the released SRF could disrupt tumor antioxidative defense to further amplify oxidative damage. Such a highly efficient and extremely low-toxic system is believed as a very promising candidate for future clinical translation.

## Materials and methods

### Materials

The source and purity of the involved reagents (used as received) are presented in Additional file 1. All the reagents if not specified were analytically pure.

### Synthesis and characterization of BCFe@SRF nanosystems

The synthesis procedures of the designed nanosystem were described step by step in Additional file 1, adopting two simple steps according to previous reports with minute modifications [28, 55]. BC and BCFe were also fabricated as control using similar approaches without the addition of ferritin and SRF (BC: without ferritin and SRF; BCFe: without SRF). In this work, the final concentration applied for the following experiments was measured basing on Ce6 concentration. The characterization approaches of the physicochemical properties, photornetrics, drug release profile and iron cycle analysis were clearly elaborated in Additional file 1.

### In vitro experiments

The murine hepatoma cell line hepa 1–6 and the mouse embryonic fibroblast cell line NIH 3T3 were utilized in this study to analyze the in vitro performance of BCFe@SRF. The cultured conditions of cells were according to our previous reports [7]. Notably, to imitate tumor hypoxia environment, the cells were placed in an anaerobic incubator with 2.5% O_2_. If not specially mentioned, different formulations were given at an equivalent dosage of 1 μM Ce6. In the in vitro assays, a 670 nm laser with power intensity of 50 mW·cm^−2^ (5 min) was utilized as PDT exciting light. To analyze the PDT effects in hypoxic environment, the cells were coated with an oil layer when exposed to laser irradiation. After laser irradiation, the cells were incubated for another 24 h and then examined by the indicated methods if not specified. The in vitro toxicity[56], intracellular localization [8], PDT outcomes [8], synergistic PDT and ferroptosis outcomes and the mechanism of anticancer effects [28, 29] were evaluated using previously reported methods, which were detailedly described in Additional file 1.

### In vivo experiments

Male BALB/c-nude mice (3–5 weeks) purchased from Wushi Laboratory Animal Co. Ltd. (China) were employed in the in vivo assays. All the formulations (at an equivalent dosage of 4 mg·kg^−1^ Ce6) were intravenously injected into the mice. After 6 h of injection, a 670 nm laser at a power intensity of 0.4 W·cm^−2^ (5 min) was employed to operate the PDT procedures. The tumor volume and mouse body weight were recorded during the indicated treatment. The histopathology, immune-histochemical analysis and protein expression of the treated tumors were also analyzed. The detailed procedures were presented point by point in Additional file 1.

## Results and discussion

### Synthesis and characterization of BCFe@SRF

In this work, a hypoxia-responsive protein-based nanoreactor with high tumor specificity and biocompatibility is proposed for amplified oxidation cancer therapy. Four key components are involved in the designed nanoplatforms: (1) Ce6-conjugated BSA (BSA-Ce6), widely considered as a charming protein-based platform with the huge prospect of clinical translation [55, 57, 58], providing 670 nm laser activated PDT; (2) ferritin, with the ability to catalyze TME-abundant H_2_O_2_ into ⋅OH and subsequently induce LPO, serving as a ferroptosis inducer to amplify ROS generation when co-performed with PDT; (3) SRF, with the capacity of destroying oxidative defense of cancer cells by inhibiting glutamate-cystine antiport system X_c_^−^ and then indirectly decreasing GSH/GPX4 [23, 28, 29], also acting as a ferroptosis promoter to further enhance the oxidation stress; (4) Azo, a hypoxia-responsive unit that can be cleaved by azoreductase overexpressed in hypoxic condition [52, 55], serving as the cross-linker to improve the stability and specificity of the nanoreactor.

**Fig. 2:**
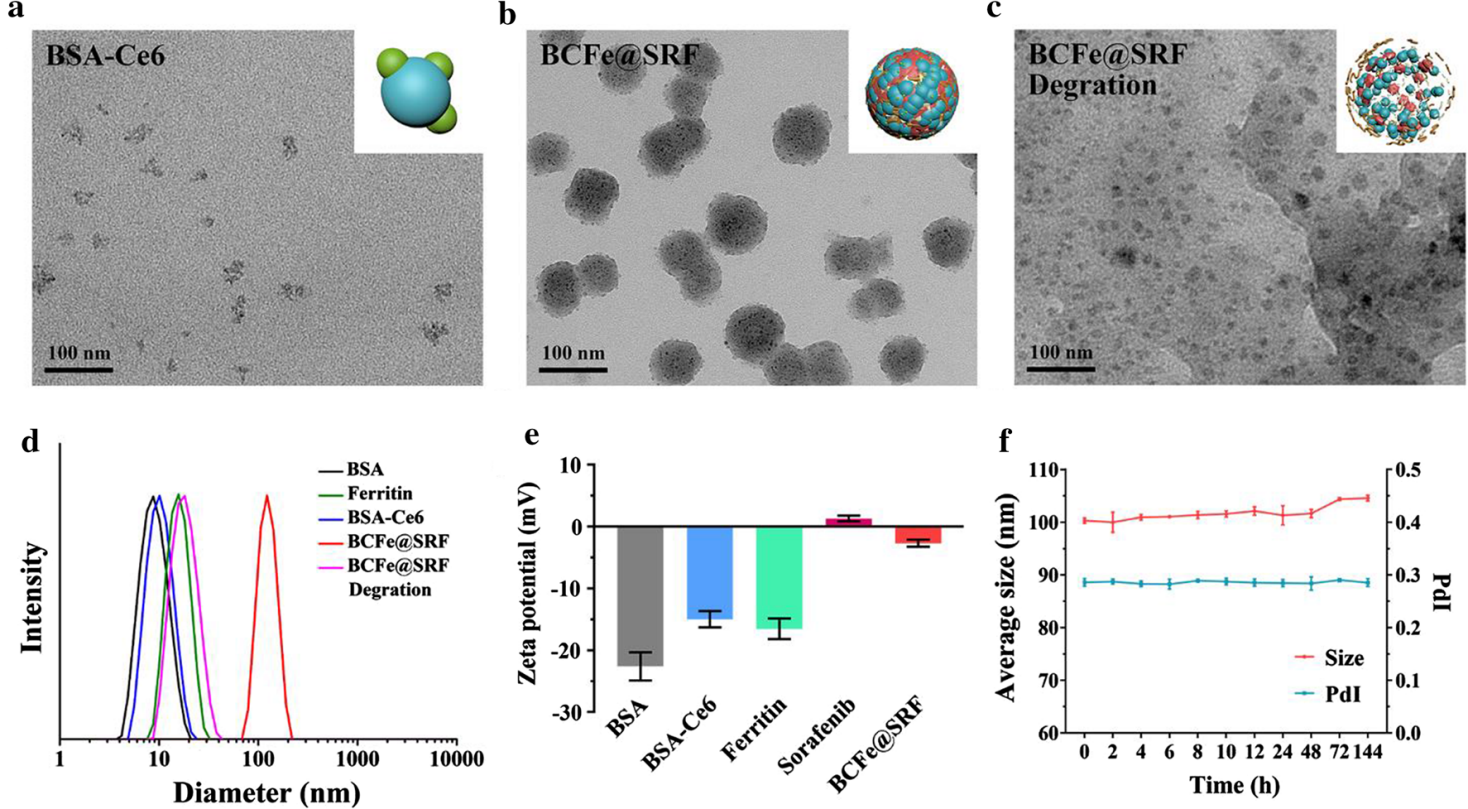
TEM images of **a** BSA-Ce6, **b** BCFe@SRF, and **c** BCFe@SRF degration product. **d** Size distribution of different formulations, final BCFe@SRF and BCFe@SRF degration product measured by DLS. **e** Zeta potential of different formulations and final BCFe@SRF determined by DLS. **f** Size distribution and PDI of BCFe@SRF in water after storing for 6 days measured by DLS

The tailored fabrication procedures of the BCFe@SRF were presented in Additional file 1: Figure S1, adopting two simple steps according to previous reports with minute modifications [28, 55]. Firstly, the Ce6 was conjugated with BSA via the formation of amide bonds to obtain BSA-Ce6 (Fig. 2a). Subsequently, the hypoxia-responsive unit Azo was employed to covalently crosslink BSA-Ce6 and ferritin, simultaneously encapsulating SRF inside the particle under hydrophobic interaction. As shown in Fig. 2b, the final product Azo-crosslinked BSA-Ce6-ferritin@SRF (BCFe@SRF) exhibited a spherical nanostructure with a diameter of 67 ± 6 nm. The dynamic light scattering (DLS) data revealed that the hydrodynamic size of BCFe@SRF was 102.6 ± 1.3 nm with polydispersity index (PDI) of 0.28 and the zeta potential changed to − 2.7 ± 0.6 mV (Fig. 2d, e, Additional file 1: Table S1), indicating the successful fabrication of BCFe@SRF. The Azo-crosslinked BSA-Ce6 (BC, without ferritin and SRF) as well as the BSA-Ce6-ferritin (BCFe, without SRF) were synthesized as control, which presented only small discrepancies of particle size and zeta potential compared with BCFe@SRF (Additional file 1: Table S1, Figure S2). The hydrodynamic size and PDI of BCFe@SRF showed no great change after storing for 6 days (Fig. 2f), confirming the stable dispersity of BCFe@SRF. Next, the loading capacities of Ce6 and SRF in BCFe@SRF were evaluated to be approximately 3.8wt% and 2.5wt% using the calibration curve by UV–Vis absorption and high performance liquid chromatography (HPLC), respectively (Additional file 1: Figure S3). The Fe content in BCFe@SRF was calculated to be 0.35 wt% detected by inductively coupled plasma-optical emission spectrometry (ICP-OES). To verify the hypoxia responsiveness of BCFe@SRF, the NP was treated with sodium dithionite (Na_2_S_2_O_4_), a chemical mimic of the hypoxia biomarker azoreductase [52], and the morphology and size distribution of degration product were evaluated by transmission electron microscopy (TEM) and DLS. The changeable structure (Fig. 2c) and decreased hydrodynamic size (Fig. 2d, 30.9 ± 2.1 nm) of BCFe@SRF demonstrated its reductase-enzyme-triggered disassembly property, suggesting that it has enormous potential for using as hypoxia-responsive drug release vehicles.

**Fig. 3:**
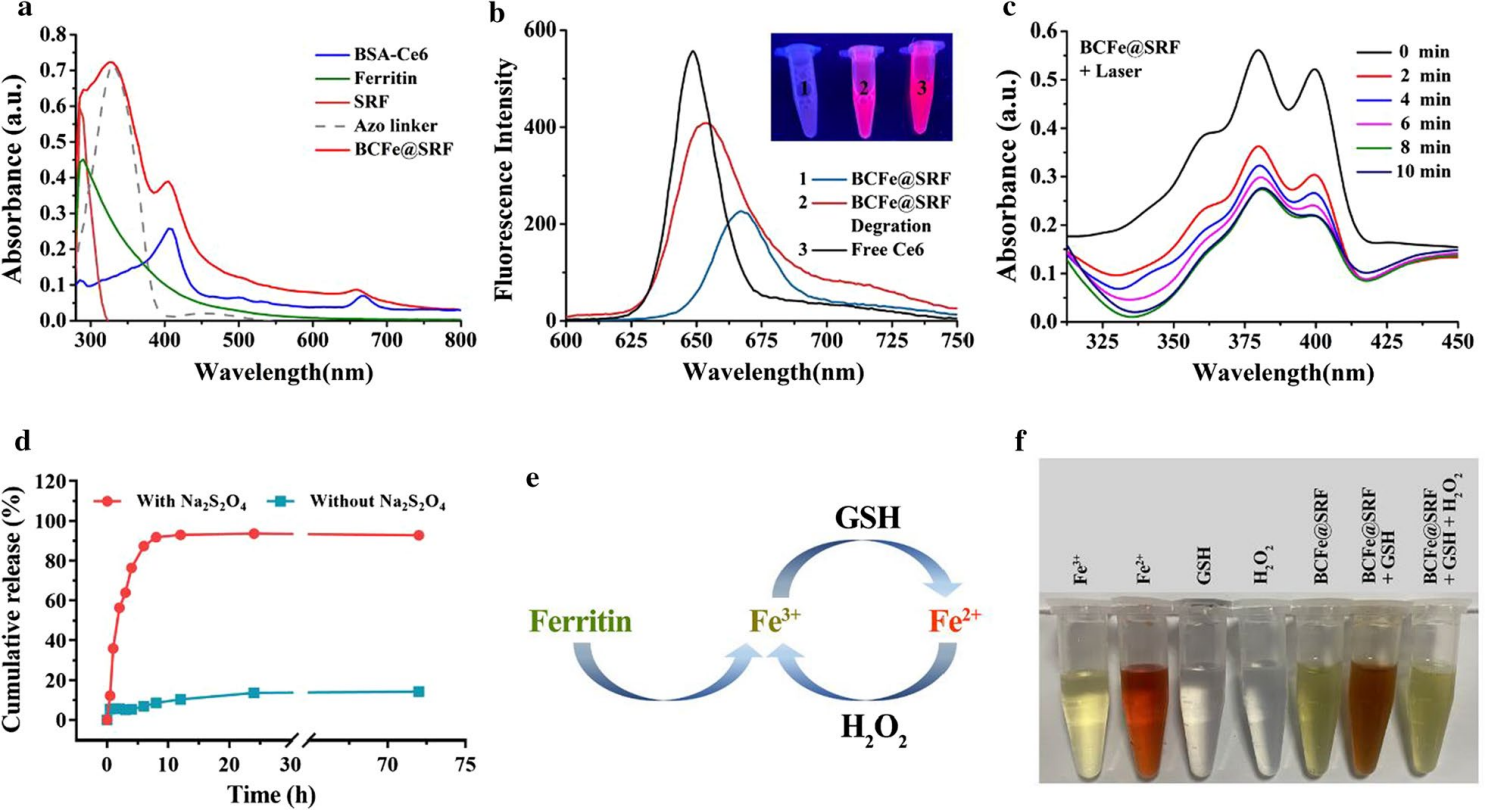
**a** UV–Vis absorption spectra of the feeding formulations and the final product BCFe@SRF. **b** Fluorescence emission spectra of free Ce6, BCFe@SRF and BCFe@SRF degration product excited by 404 nm laser. Insert: the corresponding fluorescence images. **c** The spectra of ABDA absorbance treated with BCFe@SRF upon different times of laser irradiation (670 nm, 50 mW·cm^−2^). **d** SRF release profiles from BCFe@SRF in PBS with or without Na_2_S_2_O_4_ at 37 °C. **e** The schematic diagram of the iron redox cycling. **f** Observation on the iron redox cycling of BCFe@SRF

Then, the UV–Vis absorption of BCFe@SRF was analyzed. As shown in Additional file 1: Figure S4, the absorption signals ascribing to Ce6 (380–440 nm and 640–700 nm) and BSA (280–295 nm) were clearly shown in the spectrum of BSA-Ce6, confirming the successful linking between BSA and Ce6. A new broad absorption band including characteristic peaks of ferritin, SRF and Azo (ranging from 280 to 385 nm) appeared in the spectrum of BCFe@SRF, which also demonstrated the successful fabrication of BCFe@SRF, consistent with the results of TEM and DLS analysis (Fig. 2b, d). The UV–Vis absorption spectra of BC and BCFe containing characteristic peaks of the feeding formulations also confirmed their successful preparations (Fig. 3a, Additional file 1: Figure S4). Meanwhile, the fluorescence of BCFe@SRF from Ce6 with a little redshift obviously decreased compared with free Ce6, which was mainly due to the aggregated fluorescence quenching [55], and it could be mostly recovered after BCFe@SRF degration (Fig. 3b). Then, the ROS indicator 9,10-anthracenediylbis (methylene) dimalonic acid (ABDA) was employed to evaluate the light-triggered ROS production of BCFe@SRF [8]. In this work, a 670 nm laser was utilized to trigger Ce6-based PDT as widely reported [8, 52]. Evident decrements of ABDA absorbance (350–425 nm) after laser irradiation (50 mW·cm^−2^) were observed in BCFe@SRF treated samples (Fig. 3c), whereas only slight reductions could be seen in pure BCFe@SRF or laser treated samples (Additional file 1: Figure S5), indicating the laser-dependent ROS generation ability of BCFe@SRF.

Next, the hypoxia-responsive drug release property of BCFe@SRF was examined utilizing Na_2_S_2_O_4_ as azoreductase simulant. The amounts of released drug from BCFe@SRF were quantitatively analyzed using the standard curve of SRF measured by HLPC (Additional file 1: Figure S3). As shown in Fig. 3d, no more than 15% of SRF was released from BCFe@SRF without Na_2_S_2_O_4_ treatment, demonstrating the stability of BCFe@SRF; while, in the addition of Na_2_S_2_O_4_, the release amount of SRF dramatically increased (more than 90%), suggesting the hypoxia-responsive drug release behavior of BCFe@SRF. Besides Ce6 and SRF, ferritin is another vital functional component in BCFe@SRF for the oxidation therapy. As reported, ferritin can be intracellularly degraded to release Fe^3+^, which can be reduced to Fe^2+^ by GSH [46], and then triggers the Fenton reaction to convert H_2_O_2_ into ·OH, simultaneously achieving Fe^3+^ recycle (Fig. 3e). *O*-phenanthroline was employed as a Fe^2+^ indicator, which could especially react with Fe^2+^ to form orange complexes [29]. After extracting Fe^3+^ from ferritin [59], the tinct of ferritin solution presented yellowish, in keeping with the Fe^3+^ control solution (Fig. 3f, Additional file 1: Figure S6). When GSH was added to transduce Fe^3+^ towards Fe^2+^, the solution turned orange. Additionally, the solution color rapidly faded when H_2_O_2_ was introduced, suggesting the regeneration of Fe^3+^. The phenomena confirmed the iron cycling of BCFe@SRF and ferritin when reacted with GSH and H_2_O_2_. All the above results demonstrated that the BCFe@SRF could serve as a promising therapeutic agent for designed PDT and ferroptosis synergistic treatment.

### In vitro anticancer effects of the PDT and ferroptosis synergistic therapy mediated by BCFe@SRF

**Fig. 4:**
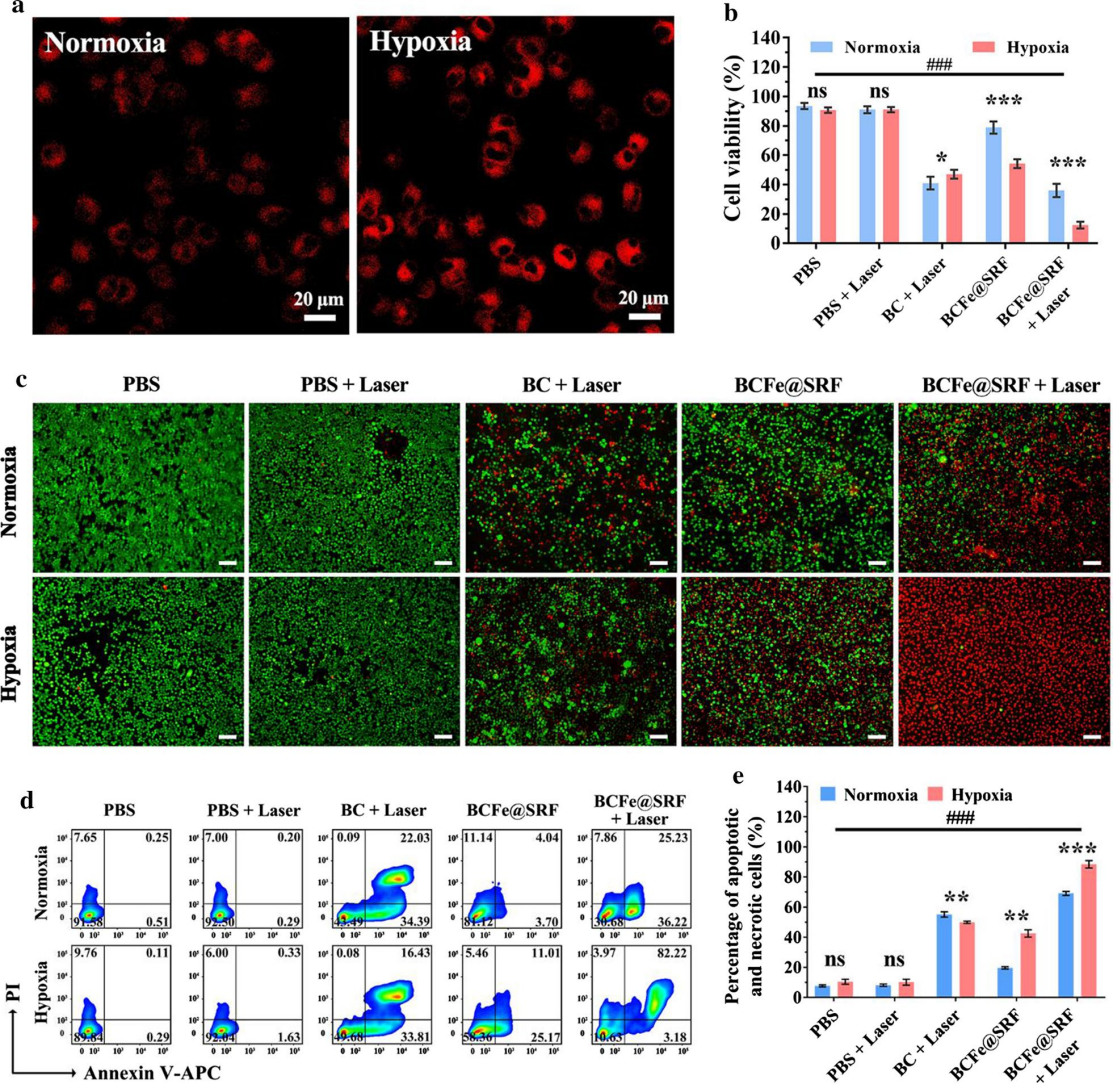
**a** CLSM images of hepa 1–6 cells treated with BCFe@SRF (Ce6 concentration: 1 μM) for 4 h in normoxic or hypoxia condition (Ce6: red, 405 nm laser excitation). **b** CCK-8 cell viability assay of hepa 1–6 cells treated with BCFe@SRF (Ce6 concentration: 1 μM) mediated PDT (670 nm light, 50 mW·cm^−2^, 5 min) in normoxic or hypoxia condition (*p < 0.05, **p < 0.01, ***p < 0.001, pairwise comparison; ^*#*^*p* < 0.05, ^*##*^*p* < 0.01, ^###^p < 0.001, compared to the BCFe@SRF + Laser group in hypoxia condition, n = 5). **c** Live/dead staining assay (green: live cells; red: dead cells) and **d** annexin V-APC/PI apoptosis assay of hepa 1–6 cells treated with BCFe@SRF (Ce6 concentration: 1 μM) mediated PDT (670 nm light, 50 mW·cm^−2^, 5 min) in normoxic or hypoxia condition. **e** Percentage of apoptotic and necrotic cells in annexin V-APC/PI apoptosis assay (*p < 0.05, **p < 0.01, ***p < 0.001, pairwise comparison; ^*#*^*p* < 0.05, ^*##*^*p* < 0.01, ^*###*^*p* < 0.001, compared to the BCFe@SRF + Laser group in hypoxia condition, n = 3)

To act as a biomedical agent, very weak cytotoxicity is a basic property of nanosystem. Therefore, the in vitro toxicity of BCFe@SRF in NIH 3T3 cells was firstly examined by CCK-8 assay. As shown in Additional file 1: Figure S7, it was found that BCFe@SRF exhibited limited cytotoxicity even at high dose, fitting the essential requirement for biological applications. The cellular internalization of BCFe@SRF in hepa 1–6 cells was then analyzed by confocal laser scanning microscopy (CLSM). As shown in Additional file 1: Figure S8, the fluorescence signals of Ce6 within cells increased over time, indicating the cellular uptake behaviors of BCFe@SRF. Notably, when the cells were cultured with BCFe@SRF in hypoxia condition, obvious signal enhancement could be observed owing to the recovery of Ce6 fluorescence after BCFe@SRF degradation (Fig. 4a), which may result from the cleavage of Azo by highly expressed reductases in hypoxia condition [52, 55]. In addition, TfR1 is overexpressed in hepa 1–6 cells (Additional file 1: Figure S9), so that it could be efficiently targeted by BCFe@SRF due to specific recognition between ferritin and TfR1. As shown in Additional file 1: Figure S10, when the cells were simultaneously (Ferritin + BCFe@SRF group) or aforehand (Pre ferritin + post BCFe@SRF group) treated with excessive free ferritin, the BCFe@SRF internalization was significantly inhibited due to the competitive inhibition or surface receptor blocking. All these demonstrated that our designed BCFe@SRF has high targeting affinity to TfR1-overexpressed tumors.

After confirming the biocompatibility and cellular uptake capacity of BCFe@SRF, its in vitro anticancer effects were then verified. In the in vitro experiments, hepa 1–6 cells were co-incubated with different formulations for 4 h and then subsequently to 670 nm light irradiation (50 mW·cm^−2^, 5 min) in normoxic or hypoxia condition and finally examined after another 24 h of incubation. To select an appropriate concentration for the following studies, BC (without ferritin and SRF) was engaged in CCK-8 assay to evaluate the pure PDT effects in normoxic circumstance. As shown in Additional file 1: Figure S11, the cell viability dramatically decreased as Ce6 concentration increased upon laser irradiation, which could be reduced to less than 40% at Ce6 concentration of 1 μM. Taking both bioavailability and efficacy into consideration, this concentration was chosen for the following studies. Subsequently, the therapeutic outcomes of synergistic PDT and ferroptosis therapy mediated by BCFe@SRF were analyzed by CCK-8, live/dead staining and annexin V-APC/PI apoptosis assays. In the CCK-8 assay, PBS and laser exhibited almost no cytotoxicity towards hepa 1–6 cells, and BCFe@SRF cultured cells in normoxic condition also showed high cell viability (78.9%) (Fig. 4b). Comparing with the normoxic condition, hypoxia cause distinct cell death of BCFe@SRF treated cells (53.6% cell viability), owing to the killing effects of released ferritin and SRF. In normoxic condition, when the BC (41.0%) or BCFe@SRF (36.0%) treated cells were exposed to light irradiation, the cell viability underwent a significant decrease ascribing to the PDT induced apoptosis. Additionally, it was noteworthy that the cell viability further decreased to 12.4% when the BCFe@SRF treated cells were cultured and irradiated under hypoxia condition, while the cell viability of BC treated cells only decreased to 47.1%. Although the hypoxia would compromise the PDT effects, the degradation of Azo-crosslinked nanosystem that makes more Ce6 exposed to laser irradiation could enhance the therapeutic efficacy to a certain extent. In addition, the killing effects could be further reinforced by ferroptosis induced by the released ferritin and SRF, all these leading to the best efficacy of BCFe@SRF treated group with laser irradiation under hypoxia incubation. To reconfirm the in vitro anticancer effects of our designed BCFe@SRF, the live/dead staining assay was then performed to intuitively observe the cell status after BCFe@SRF mediated therapy. As shown in Fig. 4c, only a few red signals could be found in the PBS and laser groups. The BCFe@SRF induced more cell death when the cells were in hypoxic incubation. The synergistic treatment by BCFe@SRF with laser irradiation under hypoxia condition was found to be the most effective in cancer cell killing, consistent with the results of CCK-8 assay (Fig. 4b). Annexin V-APC/PI staining assay was also utilized to validate the cell viability data (Fig. 4d, e). As expected, the synergistic treatment (BCFe@SRF + Laser in hypoxia condition) caused the most severe cell death (88.4%) among all groups. All these results suggested that the designed BCFe@SRF exhibited great potential to be used as both PS and ferroptosis inducer with high efficiency to restrain the hypoxic tumor growth.

### The mechanism of in vitro anticancer effects of BCFe@SRF

**Fig. 5:**
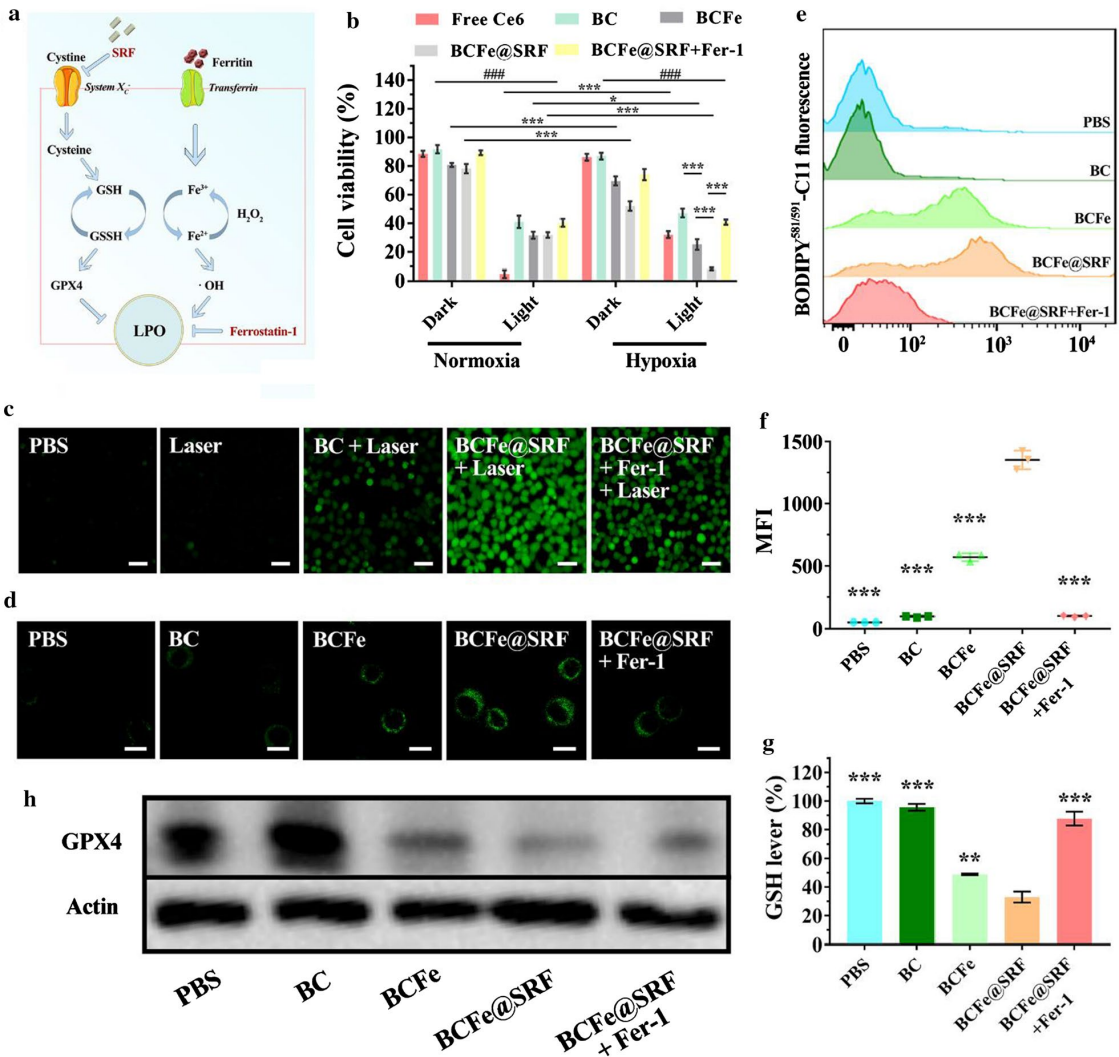
The mechanism of in vitro anticancer effects of BCFe@SRF. The hepa 1–6 cells were treated with different formulations (Ce6 concentration: 1 μM) with or without laser irradiation (670 nm light, 50 mW·cm^−2^, 5 min) in normoxic or hypoxic condition. **a** The simplified mechanism of ferroptosis. **b** CCK-8 cell viability assay (*p < 0.05, **p < 0.01, ***p < 0.001, pairwise comparison; ^*#*^*p* < 0.05, ^*##*^*p* < 0.01, ^###^p < 0.001, comparison between the same formulation in normoxic and hypoxic condition, n = 5). **c** Fluorescence microscopy images of hepa 1–6 cells treated with different formulations and ROS indicator DCFH-DA with or without laser irradiation in hypoxic condition. **d** CLSM images of hepa 1–6 cells treated with different formulations and LPO indicator BODIPY^581/591^-C11 in hypoxic condition. **e** The flow cytometry analysis of BODIPY^581/591^-C11 labeled hepa 1–6 cells treated with different formulations in hypoxic incubation. **f** The corresponding mean fluorescence intensity (MFI) of BODIPY^581/591^-C11 labeled hepa 1–6 cells analyzed by flow cytometry (*p < 0.05, **p < 0.01, ***p < 0.001, compared to the BCFe@SRF group, n = 3). **g** Intracellular GSH levels of the hepa 1–6 cells treated with different formulations (Ce6 concentration: 1 μM) in hypoxic condition. **h** Western blot analysis of intracellular GPX4 expression of hepa 1–6 cells treated with different formulations in hypoxic environment

As mentioned above, we deduced that the BCFe@SRF performed the cell killing function through PDT and ferroptosis. When BCFe@SRF was exposed to hypoxic environment within cancer cells, the highly-expressed reductases would cleave the Azo unit in BCFe@SRF to release BSA-Ce6, SRF and ferritin [52, 55], inducing ferroptosis and light-dependent PDT. Based on the established ferroptosis mechanism as illustrated in Fig. 5a, Fe^3+^ from ferritin can decrease GSH, transporting to Fe^2+^, and subsequently catalyze TME H_2_O_2_ into •OH, directly resulting in accelerated LPO generation. On the other hand, SRF can inhibit glutamate-cystine antiport system X_c_^−^ and further down-regulate GSH and GPX4 expression to destruct the cell oxidative defense system, ultimately leading to the indirect LPO promotion [23, 28, 29]. Now, the hypothetic mechanism of this BCFe@SRF mediated oxidation treatment was carefully verified.

Different formulations containing free Ce6, BC and BCFe were employed as control to treat hepa 1–6 cells under normoxic or hypoxic environment and then irradiated by 670 nm laser (50 mW·cm^−2^) for 5 min. The treated cells were analyzed after another 24 h of incubation. Ferrostatin-1 (Fer-1), a typical ferroptosis inhibitor [23, 29], was also used together with BCFe@SRF as control to investigate the mechanism. In the CCK8 assay (Fig. 5b), the cells in all groups without laser illumination under normoxic condition showed high viabilities (> 80%). Upon light irradiation, all cells underwent extremely reduced survival because of the light-triggered PDT (Free Ce6: 4.9%, BC: 41.0%, BCFe: 31.6%, BCFe@SRF: 31.8%, BCFe@SRF + Fer-1: 40.3%). In hypoxic incubation, the PDT effects of free Ce6 (32.1% of cell viability) were significantly restrained, while the outcomes of BC mediated PDT showed a lesser degree of compromise (47.1% of cell viability). This phenomenon may attribute to more Ce6 exposure to laser after Azo cleavage. Comparing the cell status of BCFe@SRF treated cells in normoxic and hypoxia condition without light illumination, more cell death could be found in hypoxic condition group (Normoxia vs Hypoxia: 78.2% vs 52.0%) owing to the ferritin and SRF induced ferroptosis. Notably, in hypoxic incubation, the BCFe@SRF treated cells upon laser irradiation showed only 8.3% of cell viability, suggesting the further enhanced anticancer effects by co-performance of PDT and ferroptosis. Moreover, in the addition of Fer-1 that could neutralize LPO, the toxicity of BCFe@SRF was obviously inhibited, further demonstrating that the BCFe@SRF mediated cell death had a great association with LPO caused oxidative damage. After evaluating the cell killing effects, the intracellular ROS production was visibly examined by 2′,7′-dichlorodihydrofluorescein diacetate (DCFH-DA) which would emit green fluorescence when reacting with ROS. The cells were cultured with different formulations for 4 h and then exposed to laser irradiation (670 nm light, 50 mW·cm^−2^, 5 min) in hypoxia condition. As shown in Fig. 5c, few fluorescence could be observed in pure PBS and Laser group, and the green signal intensity in BC + Laser group apparently got stronger owing to the PDT-induced ROS. The strongest fluorescence was observed in BCFe@SRF + Laser group, verifying the amplified ROS generation by simultaneous action of PDT and ferroptosis. As expected, when the Fer-1 was added to inhibit ferroptosis-induced ROS, some degree of signal attenuation occurred. All these results explained and reconfirmed the enhanced anticancer efficacy of synergistic PDT and ferroptosis.

After verifying the synergistic mechanism, the ferroptosis induced by BCFe@SRF was now explored in detail, with BC, BCFe and BCFe@SRF + Fer-1 as control. As reported, LPO generation, GSH and GPX4 reduction were considered as three symbolic events during ferroptosis [11, 27–29]. In all the following mechanism study assays, the cells were co-incubated with different formulations in hypoxic environment without laser irradiation. Firstly, the BODIPY^581/591^-C11 (a LPO sensor [29]) was used to examine the in vitro LPO generation by CLSM and flow cytometry (Fig. 5d–f). Owing to the contribution of ferritin-supplied LPO, the green fluorescence intensity in the cell membrane (representing LPO) in BCFe group was stronger than that in PBS or BC group. The signals were further heightened by the addition of SRF in the BCFe@SRF group, which could be greatly restrained by Fer-1. The mean fluorescence intensity (MFI) of LPO presented in BCFe@SRF group reached respectively 2.4- and 13.8-fold MFI enhancement in comparing with BCFe and BCFe@SRF + Fer-1 groups, reconfirming the cytotoxicity of BCFe@SRF involves the LPO-induced ferroptosis. Subsequently, intracellular GSH and GPX4 were respectively evaluated by Reduced GSH Assay Kit and western blot analysis. When the cells were treated with BCFe@SRF, the GSH and GPX4 expression remarkably decreased (Fig. 5g, h), implying the designed BCFe@SRF indeed destroyed the intracellular antioxidative defense system of the cancer cells. All these results verified the cytotoxicity of BCFe@SRF in hypoxia condition were associated with ferroptosis.

### In vivo anticancer effects of the PDT and ferroptosis synergistic therapy mediated by BCFe@SRF

**Fig. 6:**
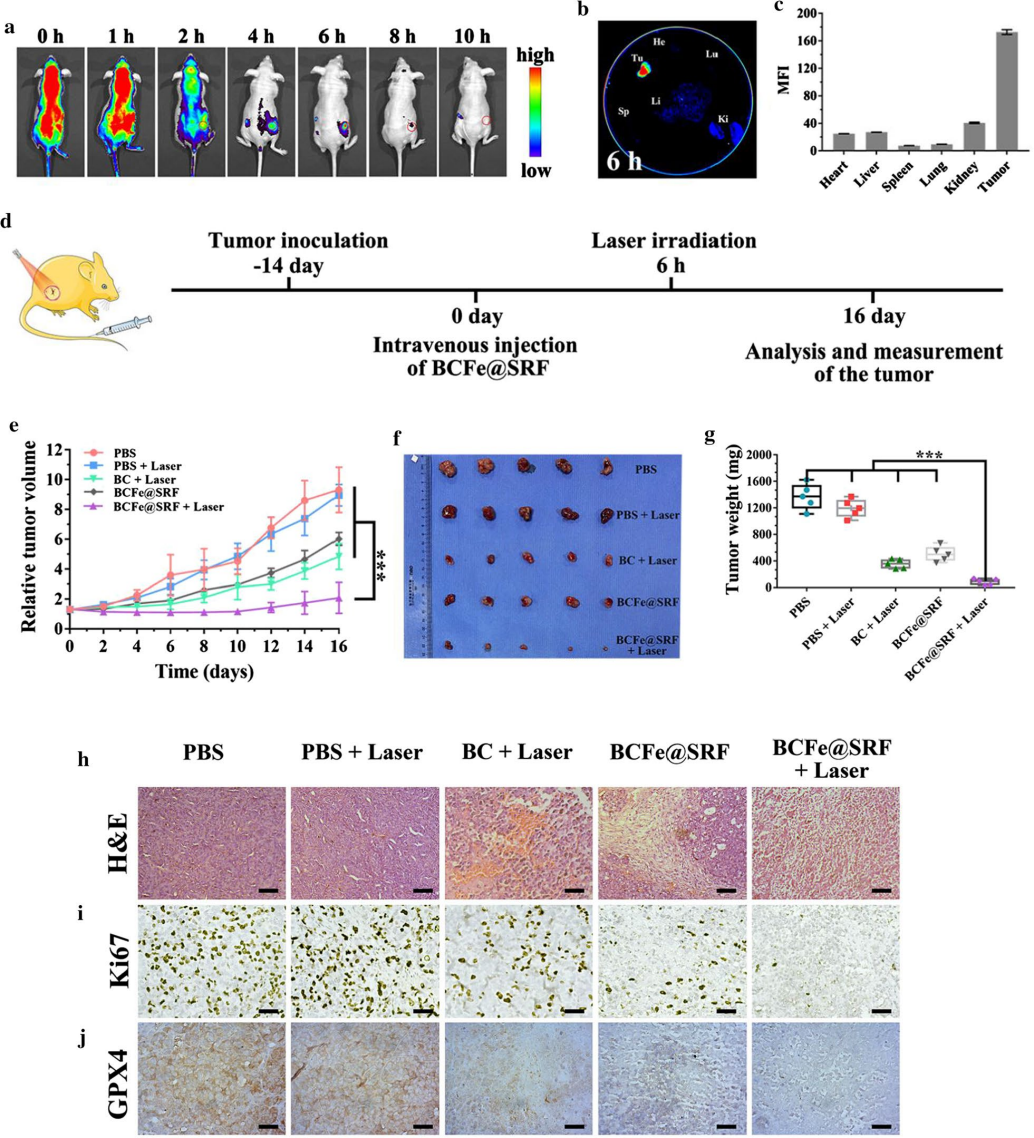
In vivo performance of BCFe@SRF in hepa 1–6 tumor bearing model (*p < 0.05, **p < 0.01, ***p < 0.001, n = 5). The involved laser irradiation (670 nm light, 0.4 W·cm^−2^, 5 min) was performed after 6 h of formulation injection. **a** In vivo fluorescence imaging of the mouse at different time points after intravenous injection of BCFe@SRF. **b** Ex vivo fluorescence imaging of the tumor and major organs harvested after 6 h of intravenous injection of BCFe@SRF. **c** The corresponding mean fluorescence intensity (MFI) of the harvested tumor and organs. **d** The schedule of the in vivo treatment for BCFe@SRF mediated synergistic therapy. **e** Time-dependent tumor growth curves. **f** The photographs and **g** average weights of the excited tumors at the end of the indicated treatment. **h** H&E, **i** Ki67 and **j** GPX4 immuno-histochemical staining of the dissected tumor after 24 h of the indicated treatment. Scale bar: 50 μm

Encouraged by the in vitro results, we next investigated the in vivo performance of our designed BCFe@SRF. First of all, the tumor bearing mouse model was established and the biodistribution of BCFe@SRF after intravenous injection was detected by tracking the Ce6 fluorescence using in vivo imaging system (IVIS). As revealed in the Fig. 6a, the Ce6 fluorescence at tumor site gradually increased along with time and actually peaked its intensity by 6 h, which demonstrated the outstanding tumor accumulation capacity of BCFe@SRF. The tumor targeting ability of such a protein-based NP mainly attributed to the passive targeting property well-known as enhanced permeation and retention (EPR) effects together with the interactions between certain proteins (albumin and ferritin) and specific receptors overexpressed on tumor cells [36–39]. Based on the IVIS observation, a mouse was sacrificed after 6 h of BCFe@SRF injection and the harvested tumor as well as major organs were then analyzed by ex vivo fluorescence imaging. It could be observed that the highest Ce6 fluorescent signals were found in the isolated tumor (Fig. 6b, c), which was concordant with the living imaging visualization (Fig. 6a). According to these results, the time point 6 h after injection was selected to conduct in vivo PDT procedure. It was worth noting that the fluorescent signals in liver was relatively weak after 6 h of BCFe@SRF injection, suggesting that some NPs can be excreted through the liver-biliary pathway [60]. More importantly, the muscle tissue and major organs are normoxic while tumor tissue is significantly hypoxic (proved by extremely high expression of hypoxia-induced factor 1α (HIF-1α) in tumor tissue, Additional file 1: Figure S12), so that the Ce6 fluorescence could recover from aggregation induced quenching in tumor tissue after BCFe@SRF degradation and showed stronger signal than that in the muscle tissue (Additional file 1: Figure S13). These results suggest that our designed BCFe@SRF exhibited hypoxia-responsive behavior to achieve highly tumor-specific therapy.

Thereafter, the in vivo antitumor effects of BCFe@SRF mediated synergistic therapy were investigated. The mice were randomly divided into five groups including PBS (as control), PBS + Laser, BC + Laser (pure PDT), BCFe@SRF (pure ferroptosis) and BCFe@SRF + Laser (synergistic therapy) groups. As illustrated in the Fig. 6d, the mice were injected with different formulations at 0 day and then the mice in laser-involved groups were exposed to laser irradiation after 6 h of injection (670 nm light, 0.4 W·cm^−2^, 5 min). The relative tumor volume and mouse body weight were recorded during another 16 days of monitoring. All the mice were sacrificed after treatment and the excised tumors were photographed and weighed to evaluate the therapeutic effects. As shown in Fig. 6e–g, the tumors in single PDT (BC + Laser) or ferroptosis therapy (BCFe@SRF) group showed a certain degree of growth suppression in comparison with those in PBS or PBS + Laser group. Additionally, more remarkable tumor inhibitions occurred in BCFe@SRF + Laser group, mainly benefiting from the amplified oxidative damage by accelerating ROS generation as well as promoting antioxidative component depletion as confirmed above (Fig. 5).

To further investigate the synergistic antitumor mechanism, one tumor-bearing mouse in each group was sacrificed after 24 h of the indicated laser irradiation procedures. The treated tumors were analyzed by H&E and Ki67 immuno-histochemical staining assays to assess the histological changes. Consistent with the tumor growth evaluation results, the most severe cell morphologic changes (cytoplasm leakages and nucleus shrinkages) and the weakest cell proliferation signals (brown granules in the cell nucleus) could be observed in the synergistic therapy (BCFe@SRF + Laser) group (Fig. 6h, i, Additional file 1: Figure S14), reconfirming the prominently preferable antitumor efficacy of our designed systems. The GPX4 expression of the harvested tumor was then evaluated by immuno-histochemical analysis. As expected, the GPX4 down-regulation occurred in the treatment groups, greatest degree of GPX4 reduction in BCFe@SRF + Laser group, indicating the compromised antioxidation ability of tumor after the indicated treatments.

Then, the potential systemic toxicity of BCFe@SRF was analyzed. First of all, during the therapeutic period, there was no apparent body weight change of the mice in all groups (Additional file 1: Figure S15), implying the bio-safety of the designed BCFe@SRF. To further verify the biocompatibility of BCFe@SRF, the major organs of one mouse in each group were collected at the end of the indicated treatment. H&E immuno-histochemical staining assay were performed to assess the histopathological changes of the major organs, and the results presented in Additional file 1: Figure S16 turned out to be that no significant physiological abnormalities appeared in all groups, reconfirming the biological security of the BCFe@SRF.

## Conclusions

In summary, a novel protein-based nanoreactor BCFe@SRF with high tumor specificity and biocompatibility was developed by covalently crosslinking BSA-Ce6 and ferritin by hypoxia-responsive unit Azo, with the SRF encapsulated inside the protein shell. The as-designed BCFe@SRF not only exhibited outstanding dispersity and stability but also could respond to hypoxic TME to release the functional components, Ce6 for laser-triggered ^1^O_2_ generation and ferritin for iron-catalyzed ·OH generation to  induce LPO based ferroptosis. Meanwhile, together with the directly amplified ROS production, the LPO accumulation could be indirectly promoted by destroying the antioxidative defense system of tumor cells through SRF-induced GSH and GPX4 reduction, which could further increase the oxidative stress within tumor sites. With excellent tumor accumulating capacity and high specificity, BCFe@SRF presented remarkable therapeutic outcomes both in vitro and in vivo. Therefore, the BCFe@SRF, integrating PDT and ferroptosis into one nanosystem with hypoxia responsiveness, offers a powerful strategy through multiple ROS enlargement cascaded pathways for future tumor-specific oxidation treatment.

## Supplementary Information

**Additional file 1. **Additional Information includes detailed materials and methods, a schematic diagram of NP fabrication, additional characterization data of different formulations and final product, NP cytotoxicity, cellular uptake of NPs, TfR1 expression of cells, PDT effects of BC, HIF-1α immunofluorescence staining, in vivo fluorescence imaging of the mouse, immuno-histochemical staining of the tumor and major organs, and mean body weights of the mice.

## Data Availability

All data generated or analysed during this study are included in this published article (and its supplementary information files).
