# Supplementary material for: Hypoxia-responsive nanoreactors based on self-enhanced photodynamic sensitization and triggered ferroptosis for cancer synergistic therapy
Source: J Nanobiotechnology. 2021 Jul 8;19:204. doi: 10.1186/s12951-021-00952-y (PMC8265128; doi:10.1186/s12951-021-00952-y)
Supplement: Supplementary file 1 — Additional file 1. Additional Information includes detailed materials and methods, a schematic diagram of NP fabrication, additional characterization data of different formulations and final product, NP cytotoxicity, cellular uptake of NPs, TfR1 expression of cells, PDT effects of BC, HIF-1α immunofluorescence staining, in vivo fluorescence imaging of the mouse, immuno-histochemical staining of the tumor and major organs, and mean body weights of the mice. [file 12951_2021_952_MOESM1_ESM.docx]

**Additional Information**

**Hypoxia-responsive nanoreactors based on self-enhanced photodynamic sensitization and triggered ferroptosis for cancer synergistic therapy**

*Xiaoyan Wang, Ming Wu, Xiaolong Zhang, Feida Li, Yongyi Zeng, Xinyi Lin*, Xiaolong Liu*, and Jingfeng Liu**

X. Wang, F. Li, Prof. X. Liu*, Prof. J. Liu*

School of Life Sciences, Fujian Agriculture and Forestry University, Fuzhou 350002, P.R. China

X. Wang, M. Wu, X. Zhang, F. Li, Y. Zeng, X. Lin^*^, Prof. X. Liu*, Prof. J. Liu*

The United Innovation of Mengchao Hepatobiliary Technology Key Laboratory of Fujian Province, Mengchao Hepatobiliary Hospital of Fujian Medical University, Fuzhou 350025, P. R. China

X. Wang, M. Wu, X. Zhang, F. Li, Y. Zeng, X. Lin^*^, Prof. X. Liu*, Prof. J. Liu*

Mengchao Med-X Center, Fuzhou University, Fuzhou 350116, P. R. China

X. Wang, F. Li, Prof. X. Liu*, Prof. J. Liu*

Fujian Institute of Research on the Structure of Matter, Chinese Academy of Sciences, Fuzhou, 350002, China

^*^ Corresponding authors: E-mail: lxy.1210@163.com (X. Lin), xiaoloong.liu@gmail.com (X. Liu) and drjingfeng@126.com (J. Liu)

**Table of Contents**

[Materials 3](#_Toc75165663)

[Synthesis of BCFe@SRF nanosystem 3](#_Toc75165664)

[BSA-Ce6 3](#_Toc75165665)

[BSA-Ce6-Ferritin@SRF (BCFe@SRF) 4](#_Toc75165666)

[Azo-cross-linked BSA-Ce6 (BC) and BSA-Ce6-Ferritin (BCFe) 4](#_Toc75165667)

[Characterization 4](#_Toc75165668)

[Instruments 4](#_Toc75165669)

[Quntitative analysis 5](#_Toc75165670)

[Performance evaluation of the functional components 5](#_Toc75165671)

[*In vitro* experiments 6](#_Toc75165672)

[Cell culture and cell density 6](#_Toc75165673)

[Material concentration and laser condition 6](#_Toc75165674)

[*In vitro* cytotoxicity assay 6](#_Toc75165675)

[Determination of cellular internalization of BCFe@SRF 7](#_Toc75165676)

[*In vitro* synergistic anticancer effect analysis 8](#_Toc75165677)

[The mechanism of synergistic PDT and ferroptosis therapy 8](#_Toc75165678)

[*In vivo* experiments 9](#_Toc75165679)

[Tumor model 9](#_Toc75165680)

[*In vivo* biodistribution of BCFe@SRF after intravenous injection 10](#_Toc75165681)

[*In vivo* synergistic antitumor effect assay 10](#_Toc75165682)

[Systemic toxicity of BCFe@SRF analysis 12](#_Toc75165683)

[Statistical analysis 12](#_Toc75165684)

[References 12](#_Toc75165685)

[Supplementary Figures 13](#_Toc75165686)

**Materials and methods**

# Materials

Chlorin e6 (Ce6, 93-98%), azobenzene-4,4'-dicarboxylic acid (Azo, 98%) were obtained from J&K Chemical Ltd. (Beijing, China). Bovine albumin (BSA, ≥ 98%), ferritin (≤ 125 mg ferritin/mL), glutathione (GSH), hydrogen peroxide (H_2_O_2_, 50%), 9,10-anthracenediylbis (methylene) dimalonic acid (ABDA, ≥ 90%) and 2′,7′-dichlorodihydrofluorescein diacetate (DCFH-DA, ≥ 97%) were purchased from Sigma-Aldrich Chemical Co. (St. Louis, MO). Sorafenib (SRF, 99.9%) was brought from Med Chem Express LLC. (Monmouth Junction, NJ, USA). Ferrostatin-1 (Fer-1, 99.89 %) was purchased from Selleck Chemicals (Houston, TX, USA). BODIPY^581/591^-C11 probe and cell culture products (Gibco) were obtained from Thermo Fisher Scientific (Waltham, MA, USA). *O*-phenanthroline (≥ 99%) was obtained from Aladdin Chemistry Co. Ltd. (Shanghai, China). Iron (III) chloride (FeCl_3_, ≥ 97%)，ferrous (II) chloride tetrahydrate (FeCl_2_·4H_2_O, 99%), sodium dithionite (Na_2_S_2_O_4_, ≥ 88.0%) and sodium dodecyl sulfonate (SDS, ≥88.0%) were obtained from Sinopharm Chemical Reagent Co., Ltd. (Shanghai, China). Ultrapure water (18.2 M·Ω resistivity at room temperature) used for all experiments was obtained from a Milli-Q Gradient System.

# Synthesis of BCFe@SRF nanosystem

BSA-Ce6: The immobilization of Ce6 onto BSA to obtain BSA-Ce6 was carried out *via* the reaction between the -COOH group of Ce6 and -NH_2_ group of BSA according to a previous report.[1, 2] Typically, 0.5 mg of Ce6 (0.84 μmol), 0.28 mg of EDC (1.46 μmol) and 0.18 mg of NHS (1.56 μmol) were dissolved in 200 µL of DMSO at 27℃ under magnetic stirring. After 2 h of reaction in dark condition, the mixture was added dropwise into 1 mL of BSA PBS solution (10 mg·mL^-1^) under intensive stirring and then kept in dark at room temperature overnight. Finally, the resultant solution was centrifugated at 12500 rpm for 10 min to remove possible aggregations and then ultrafiltered by a centrifugal filter device (MWCO 10 kDa) to remove free Ce6.

BSA-Ce6-Ferritin@SRF (BCFe@SRF): The Azo (1.6 mg, 5.92 μmol) was pre-activated with EDC (0.57 mg, 2.97μmol) and NHS (0.29 mg, 2.52 μmol) in 200 µL of DMSO for 2 h. Subsequently, the mixture, 30 μL of ferritin and 2.5 mg of SRF (5.38 μmol) were added dropwise into the BSA-Ce6 (6.22 mg) PBS solution obtained from the above step. After 12 h of stirring under dark condition, the final product BCFe@SRF was purified by dialyzing against water using a dialysis membrane (MWCO 3500) for 8 h.

Azo-cross-linked BSA-Ce6 (BC) and BSA-Ce6-Ferritin (BCFe): For the purpose of control experiments, BC and BCFe nanoparticles were prepared adopting the same synthesis procedures of BCFe@SRF without additions of ferritin and SRF (BC: without ferritin and SRF; BCFe: without SRF).

# Characterization

Instruments: The morphology of the products were observed by a high resolution transmission electron microscope (TEM; FEI Company, TALOS F2000) performed at 200 kV accelerating voltage. Dynamic light scattering (DLS) analysis was conducted using a Zeta Sizer (Nano ZS, Malvern, UK) to analyze the hydrous size, zeta potential, dispersity and stability of the formulations. The UV-Vis absorption measurement was conducted on a microplate reader (Spectra 206 Max M5, Molecular Devices). The fluorescent emission spectra of Ce6 in different formulations excited by 404 nm light was recorded on an Agilent Cary Eclipse fluorescence spectrophotometer.

Quntitative analysis: The amounts of conjugated Ce6 and encapsulated SRF in BCFe@SRF were calculated using a calibration curve of standards measured by UV-Vis absorption measurement and high performance liquid chromatography (HPLC), respectively. The Fe composition in BCFe@SRF was quantitatively evaluated by inductively coupled plasma-optical emission spectrometry (ICP-OES, Agilent ICP-OES 730). The quantitative analysis assays were performed after BCFe@SRF degration by Na_2_S_2_O_4_.

Performance evaluation of the functional components: The ROS production ability of BCFe@SRF was analyzed using ABDA as the indicator measured by UV-Vis spectrometer (BCFe@SRF: 0.16 mg·mL^-1^, ABDA: 2 mM). A 670 nm laser was employed as exciting light for laser-triggered ROS analysis (light source: K660E06M-2.000W, BWT Beijing Ltd.). To examine the SRF release profile from the nanoreactor under simulated hypoxia condition, the prepared BCFe@SRF was dispersed in a dialysis membrane (MWCO 3500) containing 2mM Na_2_S_2_O_4_ PBS solution, and then the dialysis bag was kept in a centrifuge tube containing 4 mL of PBS. The tube was placed in a shaking bed at 37 ℃. At determined time intervals, 2 mL of the dialysate was withdrawn, while the same volume of PBS was added into the residual solution. The amount of released SRF was using a calibration curve of standards measured by HPLC. The release profile of SRF without Na_2_S_2_O_4_ was measured as the control. To confirm the iron cycling of ferritin and BCFe@SRF, *o*-phenanthroline was employed as an indicator to verify the existence of Fe^2+^. Firstly, the iron in ferritin was isolated by dissolving ferritin in 2% SDS at 95℃ and then purified according to previously reported methods.[3] The product was dispersed in *o*-phenanthroline PBS solution, followed by the addition of GSH or GSH + H_2_O_2_.[4] FeCl_3_, FeCl_2_, GSH, H_2_O_2_ solutions were employed as control. The pH of all solutions was adjusted to weak acid condition.

# *In vitro* experiments

Cell culture and cell density: The murine hepatoma cell line hepa 1-6 and the mouse embryonic fibroblast cell line NIH 3T3 were cultured in Dulbecco’s Modified Eagle Medium (DMEM) containing 10% fetal bovine serum (Atlanta Biologicals, Lawrenceville, GA, USA) and 1% penicillin-streptomycin (Gibco BRL, Grand Island, NY, USA) in a humid atmosphere (37 ℃, 21% O_2_, 5% CO_2_). For imitating tumor hypoxia environment, the cells were also cultured in abovementioned medium but placed in an anaerobic incubator with 2.5% O_2_ when co-incubated with the nanosystems. In the following *in vitro* assays, the adoptive cell densities were 1×10^4^ (96-well plate), 1×10^5^ (24-well plate, 20 mm confocal laser dish) and 2×10^5^ (6-well plate) per well/dish, respectively.

Material concentration and laser condition: The applied formulations in all *in vitro* assays were measured by Ce6 concentration. Different formulations (at an equivalent dosage of 1 μM Ce6) were co-incubated with the cells for 4 h if not specially mentioned. The 670 nm laser with power intensity of 50 mW·cm^-2^ was utilized as exciting light in the *in vitro* assays. The cells were exposed to the laser for 5 min and then examined after another 24 h of incubation. To analyze the PDT effects in hypoxic environment, the cells were coated with an oil layer when exposed to laser irradiation.

*In vitro* cytotoxicity assay: The cytotoxicity of BCFe@SRF in NIH 3T3 cells were evaluated using Cell Counting Kit-8 (CCK-8, Dojindo Laboratories, Kumamoto, Japan) following the manufacturer's instruction. Briefly, the cells were seeded into a 96-well plate and incubated for 24 h. The culture medium was then replaced with 100 μL of fresh medium containing serial concentrations of BCFe@SRF (Ce6 concentration from 0.25 to 4 µM), and then the cells were incubated for another 24 h. After washing with PBS thrice, 10 µL of CCK-8 solution mixed with 90 µL of culture medium was added into each well and the cells were further incubated for 30 min. The absorbance intensity at 450 nm was measured at a Spectra Max M5 microplate reader. The relative cell viabilities (%) were measured from five wells in parallel and expressed as the means ± SD, calculated from the formula cell viability (%) = OD (sample)/OD (control) × 100%.

Determination of cellular internalization of BCFe@SRF: To evaluate the intracellular localization of BCFe@SRF in hepa 1-6 cells, the cells were seeded into 20 mm confocal laser dishes for 24 h and then the BCFe@SRF was added into each dish. After 1-4 h of incubation with the nanoreactors, the cells were washed with PBS, fixed with paraformaldehyde (4%) and then imaged using a confocal laser scanning microscope (CLSM; Zeiss LSM780). To confirm the intracellular Ce6 fluorescence recovery in hypoxia condition, the cells were incubated with the same concentration of BCFe@SRF in normoxic or hypoxic environment for 4 h and then also examined by CLSM. The transferrin receptor 1 (TfR1) expression in cells was analyzed by western blotting. The total protein was separated according to their size on the SDS-polyacrylamide gel and then transferred from the gel onto a nitrocellulose membrane. The membranes were incubated with primary antibody against TfR1 (Abcam, ab214039) after blocking with 5 % non-fat dry milk for 1.5 h. Followed by rinsing with antibody-buffer solution, the membranes were exposed to the HRP-conjugated secondary antibodies (Abcam, 1:5000) at room temperature for 1h. The signals were detected by Gel Doc XR imaging system (Bio-Rad, Lab, Hercules, CA). To verify the specific uptake of BCFe@SRF through interaction between TfR1 and ferritin, the cells were aforehand (pre-incubated with ferritin for 3 h) or simultaneously treated with excess free ferritin (50 μg·mL^-1^) and then examined by CLSM.

*In vitro* synergistic anticancer effect analysis: The CCK-8 viability assay was first performed to investigate the PDT effects of BC in hepa 1-6 cells. The cells in 96-well plates were incubated with various amounts of BC (Ce6 concentration from 0 to 4 µM) for 4 h and then washed with PBS buffer to remove free nanoparticles. Afterwards, the cells were cultured in fresh medium and exposed to laser irradiation for 5 min. After another 24 h of incubation, the cell viability was tested by CCK-8 assay adopting the same procedures as described above. CCK-8 assay was also performed to quantitatively evaluate the BCFe@SRF mediated synergistic anticancer effects in normoxic or hypoxic conditions, using the same procedures as described above. The final concentration applied for the experiments was measured basing on Ce6 concentration.

For qualitative analysis, the *in vitro* synergistic anticancer effect of BCFe@SRF was reconfirmed by live/dead staining and apoptosis assay.[5, 6] The method of cell treatment was the same as described above. After 24 h of the indicated treatment procedures, in live/dead staining assay, the cells seeded on 96-well plate were stained using LIVE/DEAD Viability/Cytotoxicity Kit (Invitrogen, Eugene, OR, USA)), and whereafter the cells were observed using a fluorescence microscope (Zeiss Axio Vert.A1). For apoptosis assay, the treated cells seeded on 24-well plate were stained using annexin V-allophycocyanin (APC)/propidium iodide (PI) apoptosis detection kit (KeyGEM Biotech Co., Ltd., Jiangsu, China) and finally detected by flow cytometry (FCM; BD FACSVerse).

The mechanism of synergistic PDT and ferroptosis therapy: To evaluate the mechanism of the BCFe@SRF mediated anticancer effects, the cell viability, intracellular ROS generation, LPO accumulation in cell membrane, and intracellular GSH/GPX4 content were determined. The cells were firstly treated with different formulations involving free Ce6, BC (pure PDT), BCFe (without SRF) and BCFe@SRF for 4 h. Fer-1 was employed to block ferroptosis pathway as control. To evaluate the cell viability in different groups, CCK-8 assay was performed following the abovementioned procedures with or without laser irradiation in normoxic or hypoxic conditions. For intracellular ROS visualization, after being treated with different formulations for 4 h, the cells seeded on 96-well plate were washed with PBS and cultured with fresh medium containing 40 μM of DCFH-DA probe for 30 min. After replacement with fresh culture medium (serum-free), the cells were exposed to laser irradiation for 5 min and finally visualized by a fluorescence microscope.

The fluorescence probe BODIPY^589/591^-C11 was employed to assess the LPO level in cell membrane, which can insert into lipid membranes and be oxidized by LPO. The oxidative product can emit green fluorescence when excited by 488 nm laser (λ_ex_: 485 nm, λ_em_: 520 nm). The cells seeded on 20 mm confocal laser dishes were treated with different formulations for 4 h in hypoxic condition and then incubated in serum-free medium containing 5 μM of BODIPY^589/591^-C11 for 15 min. Finally, the cells were observed by CLSM and detected by flow cytometry. For intracellular GSH detection, the cells seeded on 24-well plate were firstly treated with different formulations in hypoxic condition for 4 h. Afterwards, a Reduced Glutathione (GSH) Assay Kit (Solarbio Science﹠Technology Co., Ltd., Beijing, China) was used to determine the difference in intracellular GSH level among groups, according to the manufacturer’s instruction. The GPX4 expression in cells was determined by western blot adopting the method mentioned above except for replacing the primary antibody (anti-GPX4 (abcam, ab231174).

# *In vivo* experiments

Tumor model**:** To establish the tumor model, 1 × 10^7^ hepa 1-6 cells suspended in 100 μL of PBS were subcutaneously injected into the right flank of hind leg region of male BALB/c-nude mice (obtained from Wushi Laboratory Animal Co. Ltd., China). 14 days later, when the tumor size reached approximately 50 mm^3^, the tumor bearing mice were randomly divided into different groups.

*In vivo* biodistribution of BCFe@SRF after intravenous injection: After the mouse receiving systemic administration (intravenous injection of BCFe@SRF (Ce6: 4 mg·kg^-1^) in 100 μL of PBS), the mouse was placed in an *in vivo* imaging system (IVIS, Perkin Elmer) to monitor the fluorescent signal of Ce6 at tumor site (excited by 660 nm laser). Meanwhile, another mouse receiving the same treating procedure was sacrificed after 6 h of intravenous injection, whose tumor and major organs (kidney, lung, spleen, heart and liver) were imaged by Gel Doc XR imaging system to examine the tumor targeting ability of BCFe@SRF. To detect tumor hypoxia, HIF-1α immunofluorescence staining was carried out. Briefly, one tumor-bearing mouse was sacrificed and the harvested tumor, muscle tissue and major organs from the mouse were fixed in formalin, embedded by paraffin, and further sectioned into slices with thickness of 4 mm. Subsequently, the slices were stained with anti-HIF1α (Cell Signaling Trchnology, D1S7W) and observed by CLSM. A mouse receiving simultaneously intramuscular and intratumoral injections of BCFe@SRF (Ce6: 0.1 mg·kg^-1^) was imaged after 4 h of injection by Gel Doc XR imaging system.

*In vivo* synergistic antitumor effect assay**:** For *in vivo* synergistic anticancer effect assay, the tumor-bearing mice were randomly allocated into five groups: (a) PBS; (b) PBS+Laser; (c) BC+Laser; (d) BCFe@SRF; (e) BCFe@SRF+Laser. Details of the therapeutic method given for each group are as follows:

(1) PBS: 100 μL of sterilized PBS for each mouse;

(2) PBS+Laser: 100 μL of sterilized PBS for each mouse, followed by laser irradiation;

(3) BC+Laser: 100 μL of BC in PBS solution for each mouse, followed by laser irradiation;

(4) BCFe@SRF: 100 μL of BCFe@SRF in PBS solution for each mouse;

(5) BCFe@SRF+Laser: 100 μL of BCFe@SRF in PBS solution for each mouse, followed by laser irradiation;

All formulations (at an equivalent dosage of 4 mg·kg^-1^ Ce6) were intravenously injected into the mice in corresponding groups at day 0. After 6 h of injection, the tumor site was exposed to the laser for 5 min. 670 nm laser with power intensity of 0.4 W·cm^-2^ was employed to perform the *in vivo* laser irradiation procedure. The therapeutic effects were examined by monitoring tumor volume in each group every 2 days, up to 16 days. The tumor size was measured using an electronic caliper. The tumor volume (*V*) was calculated using the equation tumor length × (tumor width)^2^/2. Relative volume *V/V_0_* (*V_0_* as the initial tumor volume before therapy) was used to evaluate the relative tumor growth rate. Simultaneously, the body weights of the treated mice were surveyed at the same time to evaluate the side effects. All the tumors were harvested at the end of the studies for the following photographing and weighing.

To evaluate the histological changes of tumors and protein expression, one tumor-bearing mouse was sacrificed in each group after 24 h of laser irradiation. Afterward, the harvested tumors from the treated mice were fixed in formalin, embedded by paraffin, and further sectioned into slices with thickness of 4 mm. Subsequently, they were stained with hematoxylin and eosin (H&E) for histopathology evaluation and anti-Ki67 (Servicebio, GB11141) for immunohisto-chemical analysis, respectively. GPX4 protein expression was also detected using GPX4 antibody (abcam, ab203031) by immuno-histochemical analysis. The tumor slices were then imaged using a Zeiss microscope (Axio Lab.A1).

Systemic toxicity of BCFe@SRF analysis: To analyze the potential systemic toxicity of BCFe@SRF, one treated mouse was sacrificed in each group at the end of indicated treatment, and then the major organs of the mice were harvested, fixed in formalin, embedded by paraffin, sectioned into slices, stained with H&E and finally observed by a Zeiss microscope.

# Statistical analysis

All quantitative data were presented as the mean ± standard deviation (SD). Statistical analysis among different groups was performed using GraphPad Prism 7. Statistical significance (*p* < 0.05, *p* < 0.01, and *p* < 0.001) was evaluated using the unpaired two tailed Student t-test. When *p* < 0.05, there are statistical significance between the compared groups.

# References

1. Yang G, Phua SZF, Lim WQ, Zhang R, Feng L, Liu G, Wu H, Bindra AK, Jana D, Liu Z, Zhao Y. A hypoxia-responsive albumin-based nanosystem for deep tumor penetration and excellent therapeutic efficacy. Adv Mater. 2019;31:e1901513.

2. Xu T, Ma Y, Yuan Q, Hu H, Hu X, Qian Z, Rolle JK, Gu Y, Li S. Enhanced ferroptosis by oxygen-boosted phototherapy based on a 2-in-1 nanoplatform of ferrous hemoglobin for tumor synergistic therapy. ACS Nano. 2020;14:3414-25.

3. La A, Nguyen T, Tran K, Sauble E, Tu D, Gonzalez A, Kidane TZ, Soriano C, Morgan J, Doan M, Tran K, Wang CY, Knutson MD, Linder MC. Mobilization of iron from ferritin: new steps and details. Metallomics. 2018;10:154-68.

4. Liu T, Liu W, Zhang M, Yu W, Gao F, Li C, Wang SB, Feng J, Zhang XZ. Ferrous-supply-regeneration nanoengineering for cancer-cell-specific ferroptosis in combination with imaging-guided photodynamic therapy. ACS Nano. 2018;12:12181-92.

5. Lin X, Wu M, Li M, Cai Z, Sun H, Tan X, Li J, Zeng Y, Liu X, Liu J. Photo-responsive hollow silica nanoparticles for light-triggered genetic and photodynamic synergistic therapy. Acta Biomater. 2018;76:178-92.

6. Lin X, Wang X, Li J, Cai L, Liao F, Wu M, Zheng D, Zeng Y, Zhang Z, Liu X, Wang J, Yao C. Localized NIR-II photo-immunotherapy through the combination of photothermal ablation and in situ generated interleukin-12 cytokine for efficiently eliminating primary and abscopal tumors. Nanoscale. 2021;13:1745-58.

# Additional Figures


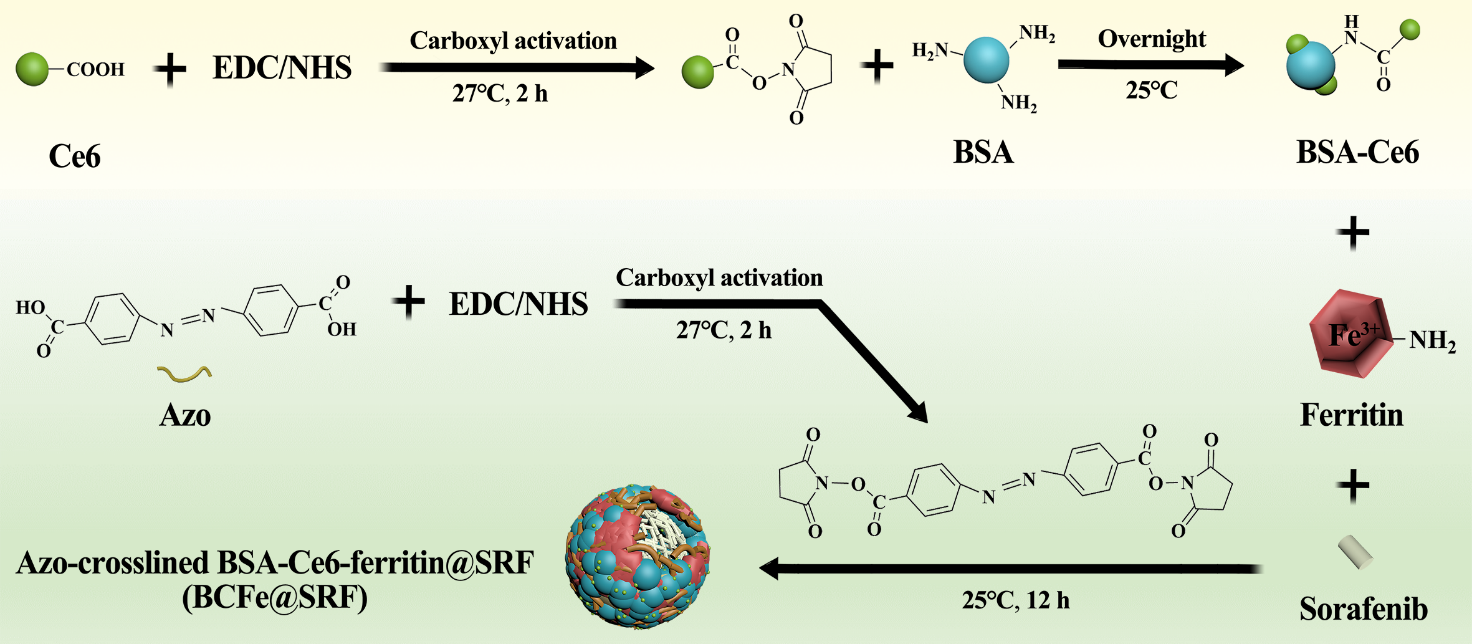


Figure S1 The schematic diagram of BCFe@SRF fabrication.

**Table S1.** Characterization of different intermediate formulations and final product measured by DLS.

| **Sample** | **Hydrodynamic**  **particle size**  **(nm)** | **PdI** | **Zeta potential**  **(mV)** |
| --- | --- | --- | --- |
| BSA | 11.4 ± 1.2 | 0.17 | -22.6 ± 2.3 |
| BSA-Ce6 | 18.0 ± 1.3 | 0.11 | -15.0 ± 1.3 |
| Ferritin | 22.7 ± 0.1 | 0.22 | -16.5 ± 1.6 |
| BCFe@SRF | 102.6 ± 1.3 | 0.28 | -2.7 ± 0.6 |
| BC | 86.0 ± 5.4 | 0.31 | -1.7 ± 0.6 |
| BCFe | 91.4 ± 5.0 | 0.18 | -1.5 ± 0.4 |


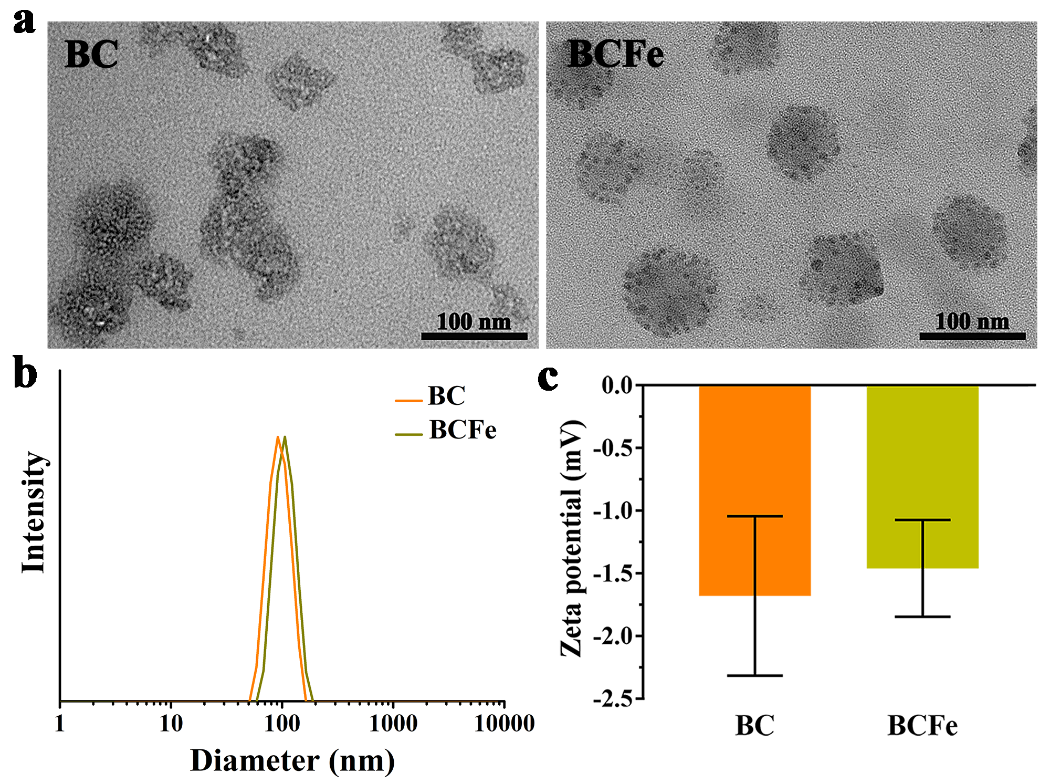


**Figure S2** (**a**) TEM images of BC and BCFe. (**b**) Size distribution and (**c**) zeta potential of BC and BCFe measured by DLS.


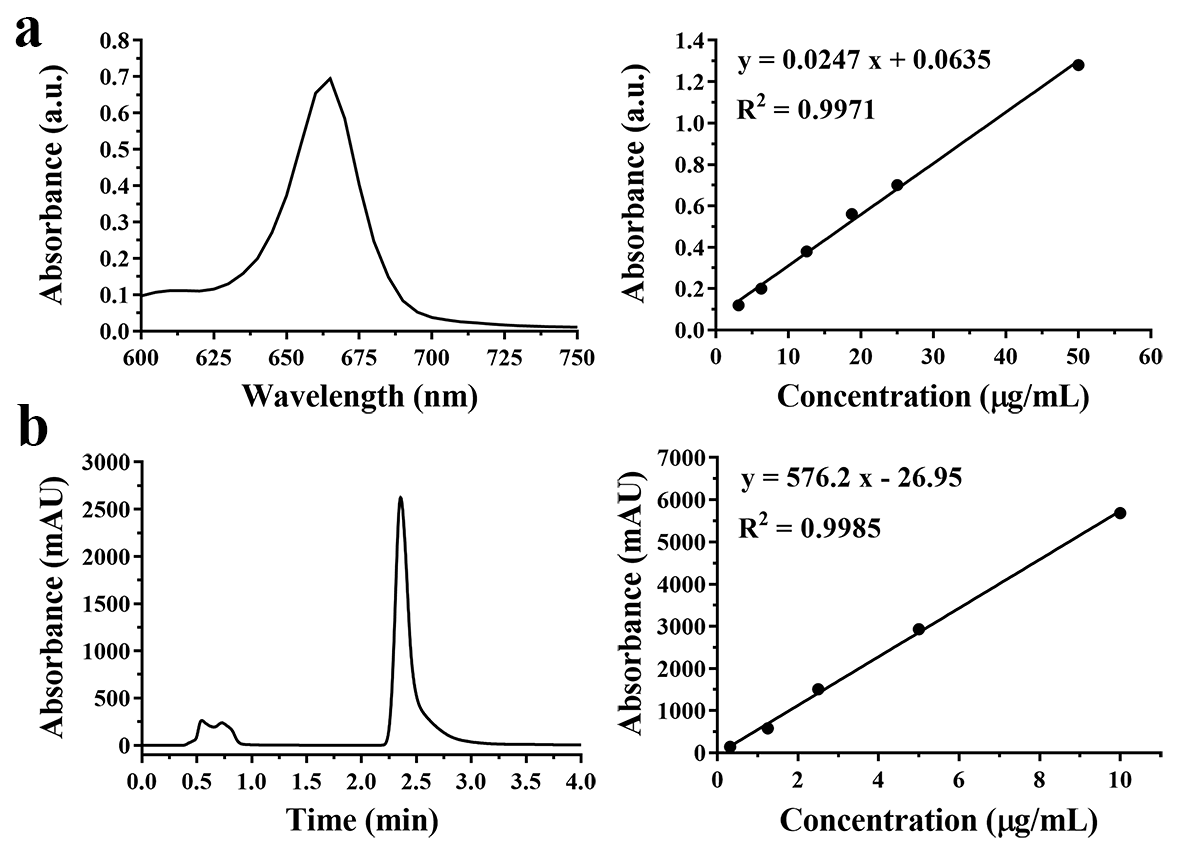


**Figure S3** (**a**) UV-Vis absorption analysis and the standard curve of Ce6 (absorbance at 670 nm). (**b**) HLPC analysis and the standard curve of SRF.


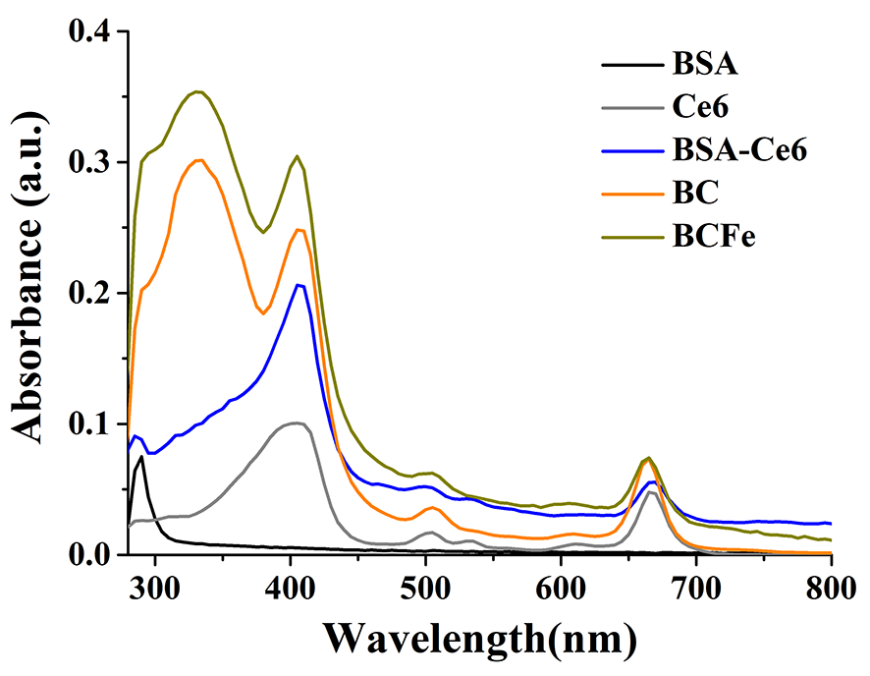


**Figure S4** UV-Vis absorption spectra of BSA, Ce6, BSA-Ce6, BC and BCFe.


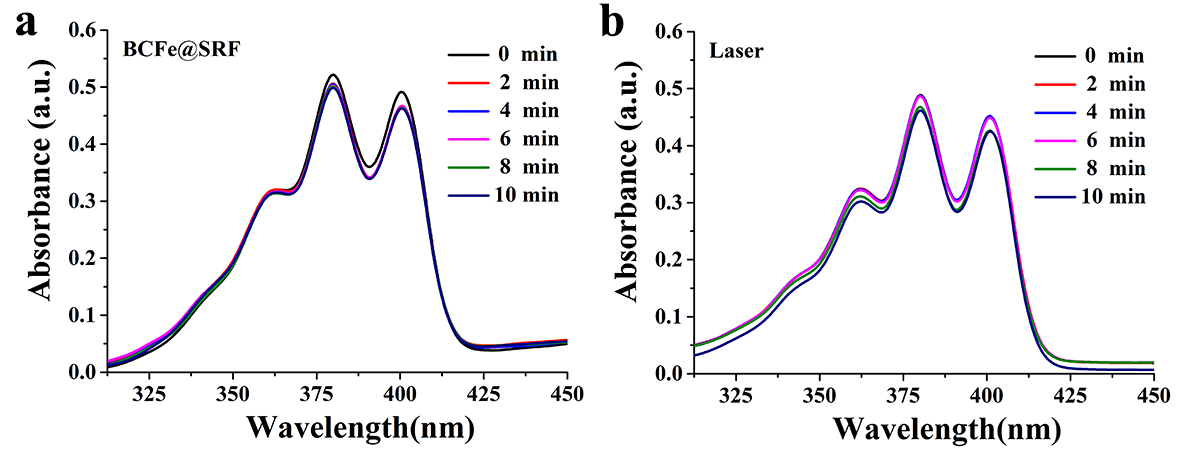


**Figure S5** (**a**) The spectrum of ABDA absorbance treated with BCFe@SRF under room light for different times. (**b**) The spectrum of ABDA absorbance treated with pure laser irradiation (670 nm laser, 50 mW·cm^-2^) for different times in the absence of BCFe@SRF.


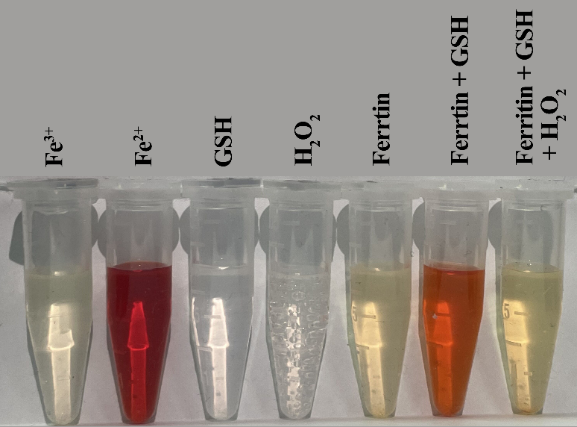


**Figure S6** Observation on the iron cycling of ferritin.


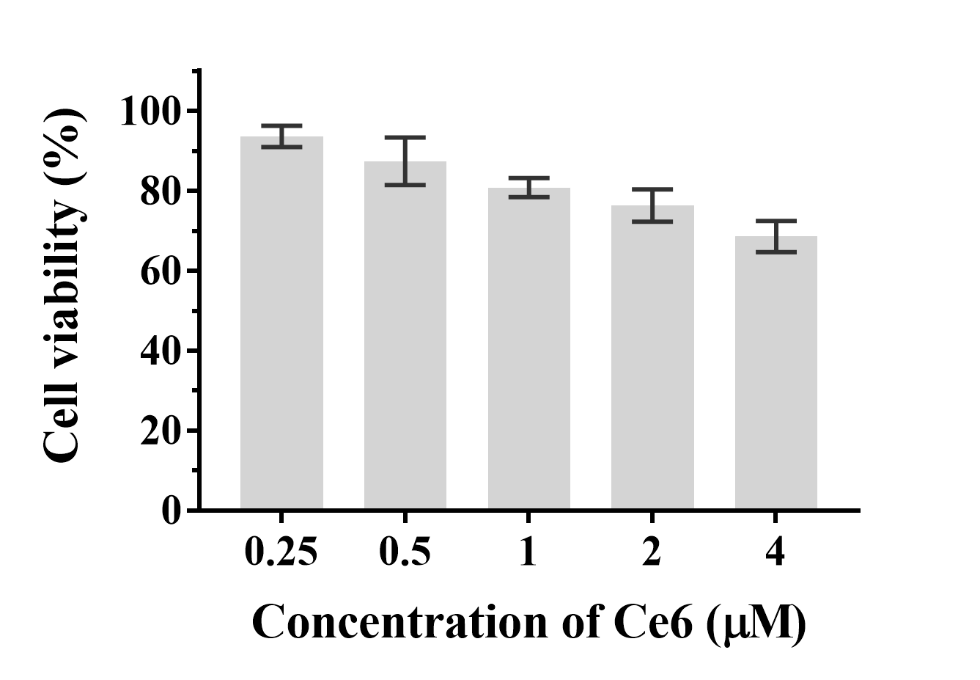


**Figure S7** CCK-8 viability assay of NIH 3T3 cells treated with BCFe@SRF at various concentrations for 24 h (mean ± SD, n = 5).

**
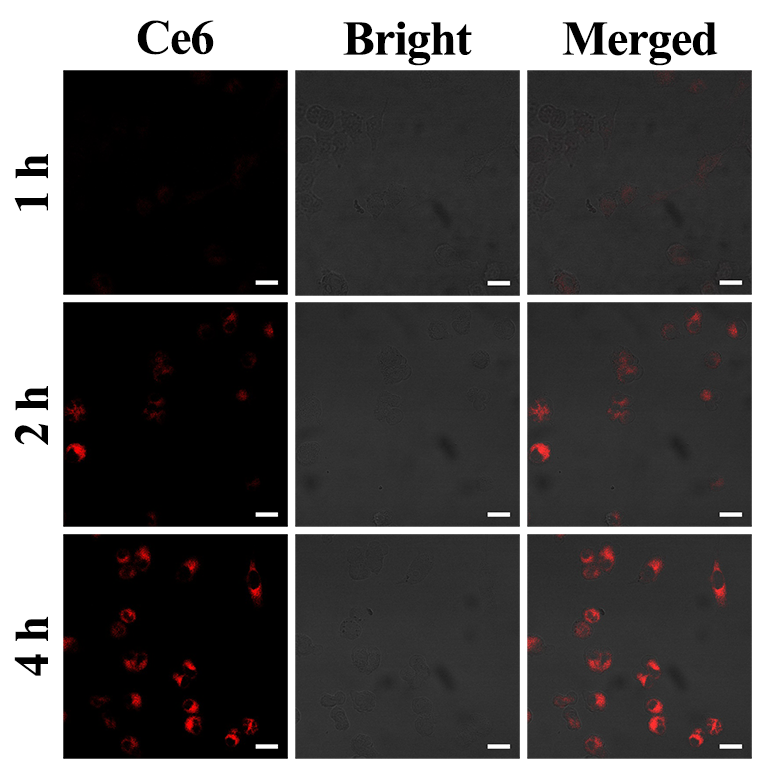
**

**Figure S8** CLSM images of hepa 1-6 cells incubated with BCFe@SRF (Ce6 concentration: 1μM) in normoxic condition for 1-4 h (Ce6: red, 405 nm laser excitation). Scale bar: 20 μm.


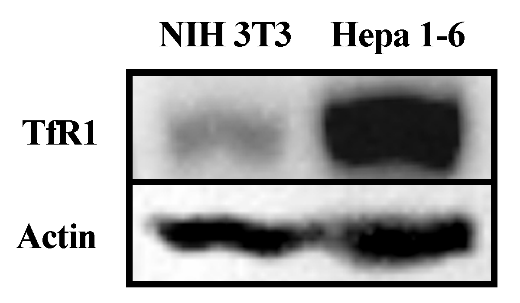


Figure S9 Western blot analysis of TfR1 expression of NIH 3T3 cells and hepa 1-6 cells.


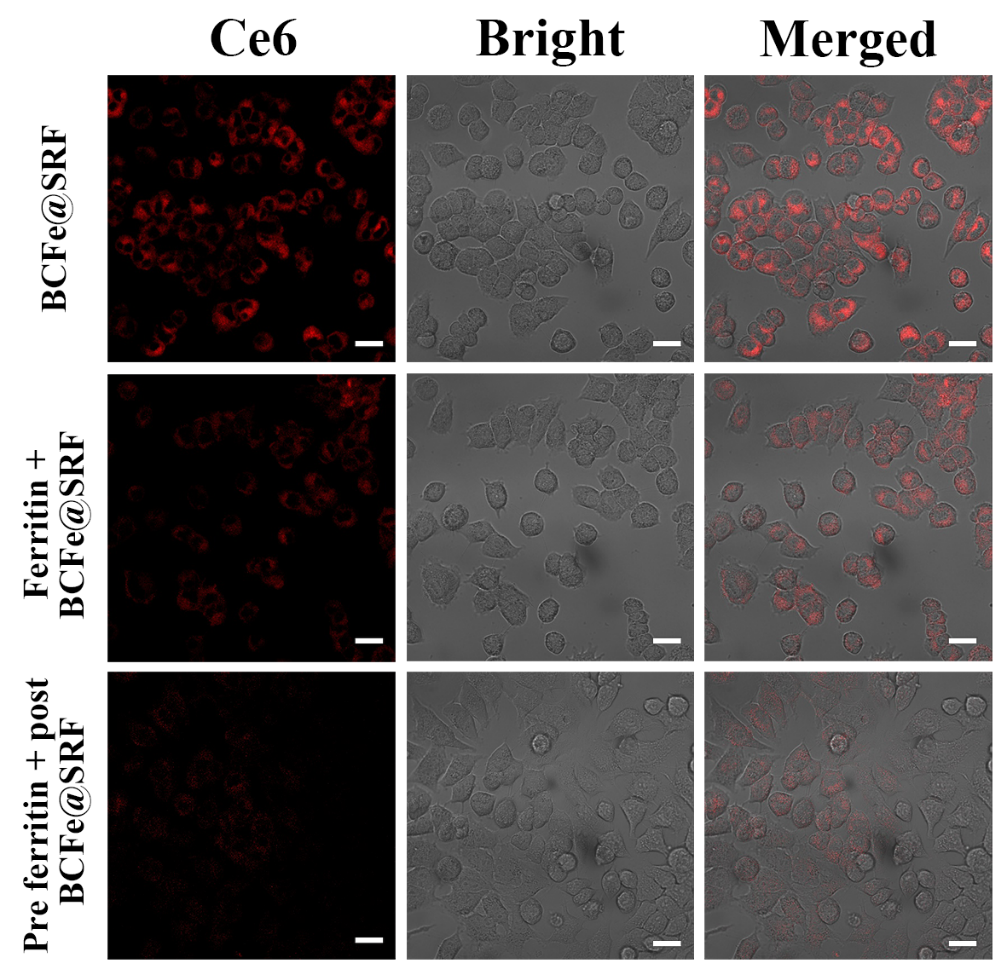


Figure S10 Uptake of BCFe@SRF by hepa 1-6 cells. Scale bar: 20 μm.


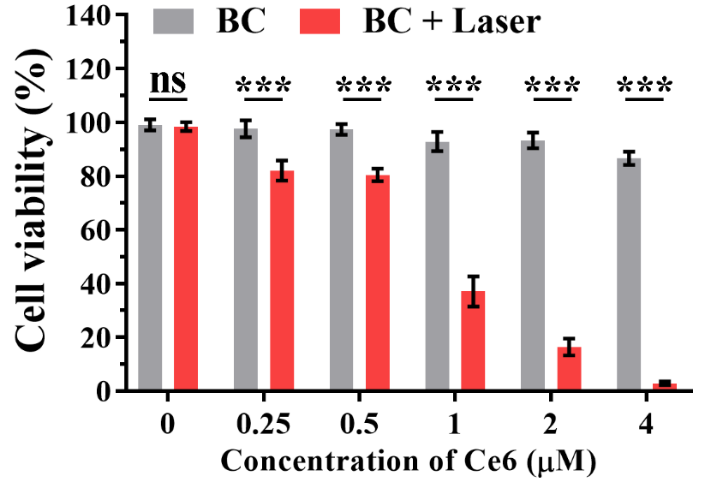


**Figure S11** CCK-8 cell viability assay of hepa 1-6 cells treated with BC mediated PDT (670 nm light, 50 mW·cm^-2^, 5 min) in normoxic condition (**p* < 0.05, ***p* < 0.01, ****p* < 0.001, n = 5).


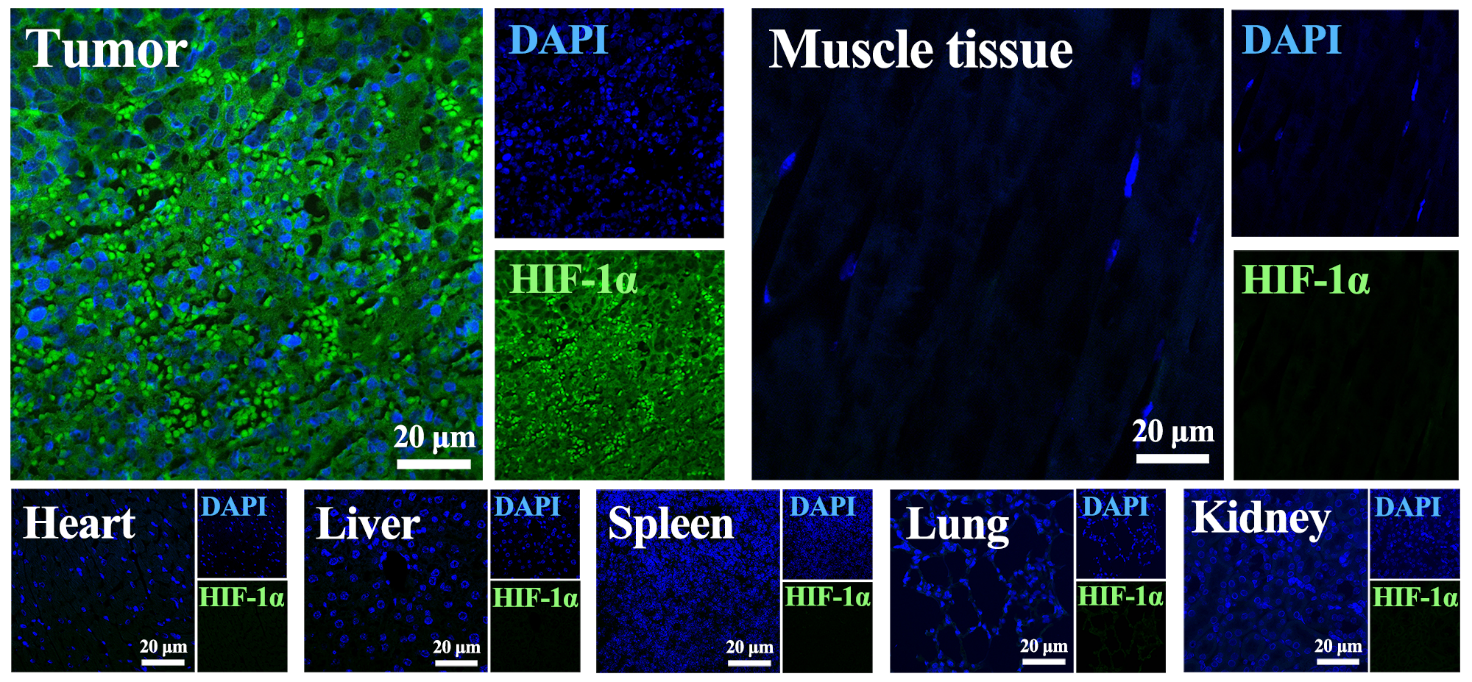


Figure S12 HIF-1α immunofluorescence staining of the tumor, muscle tissue and major organs.


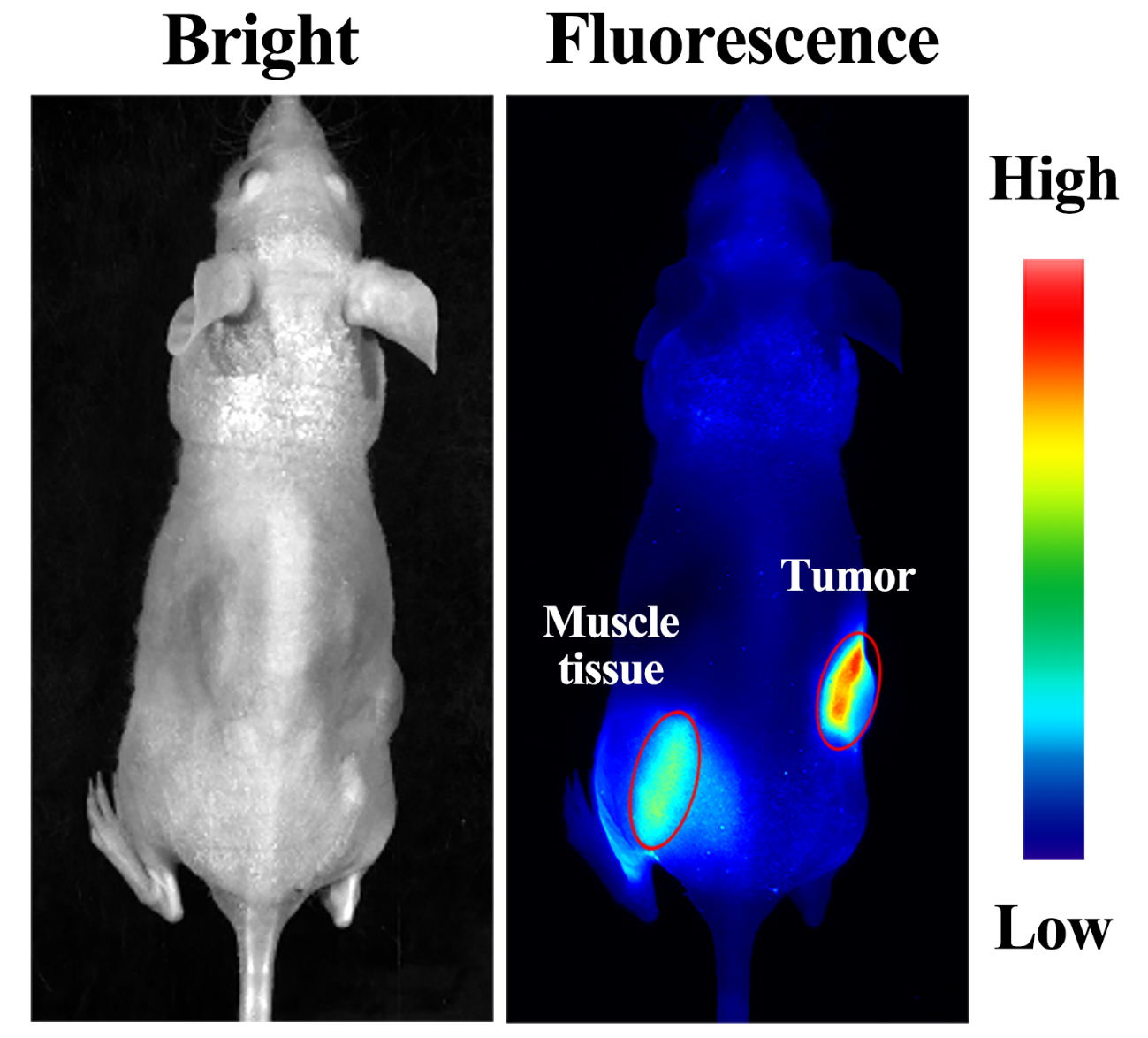


Figure S13 *In vivo* fluorescence imaging of the mouse after 4 h of intramuscular and intratumoral injections of BCFe@SRF (0.1 mg·kg^-1^ Ce6).


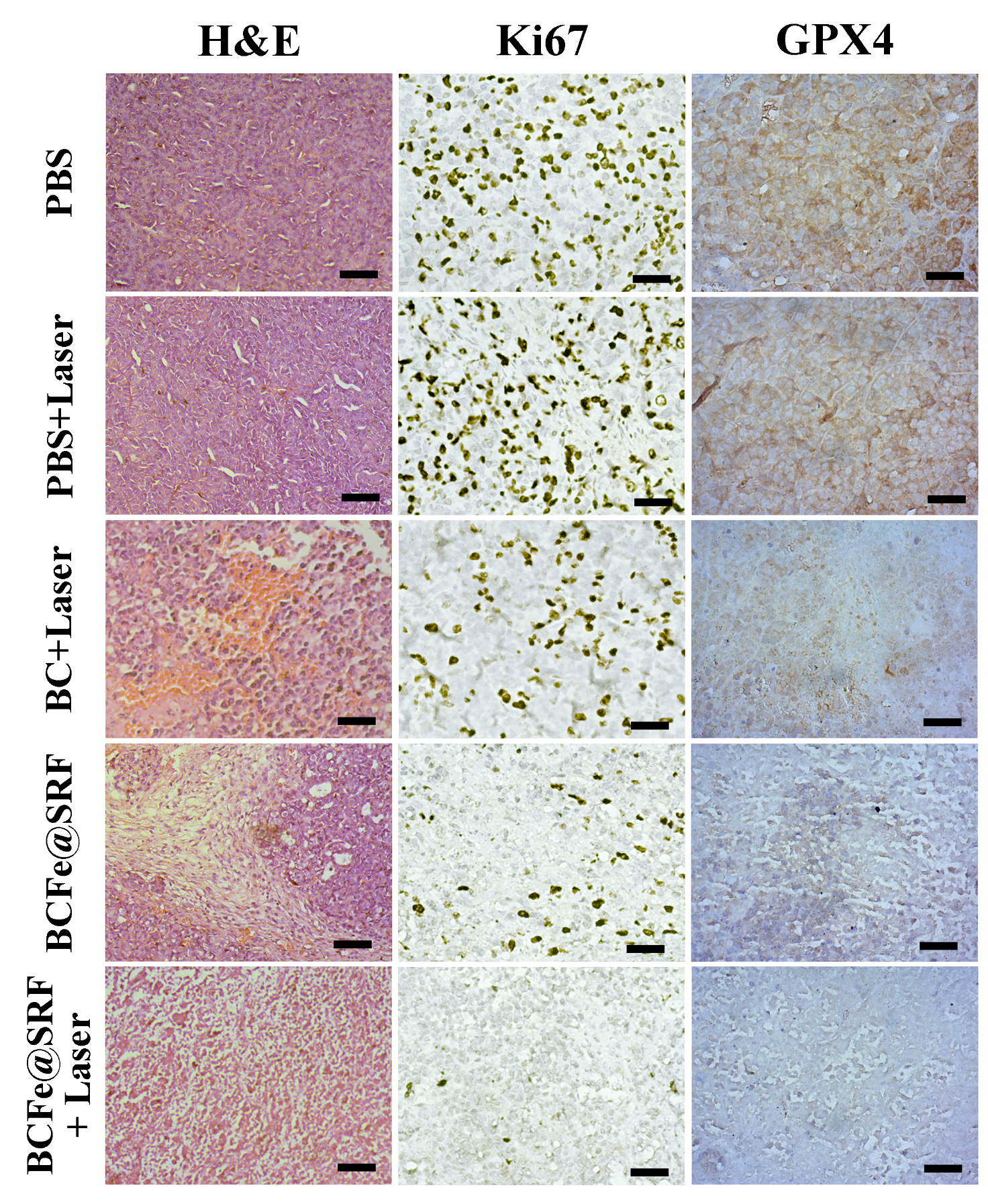


**Figure S14** H&E, Ki67 and GPX4 immuno-histochemical staining of the dissected tumor after 24 h of the indicated treatment (rearranged from Fig.6h-j for clearer visualization). Scale bar: 50 μm.


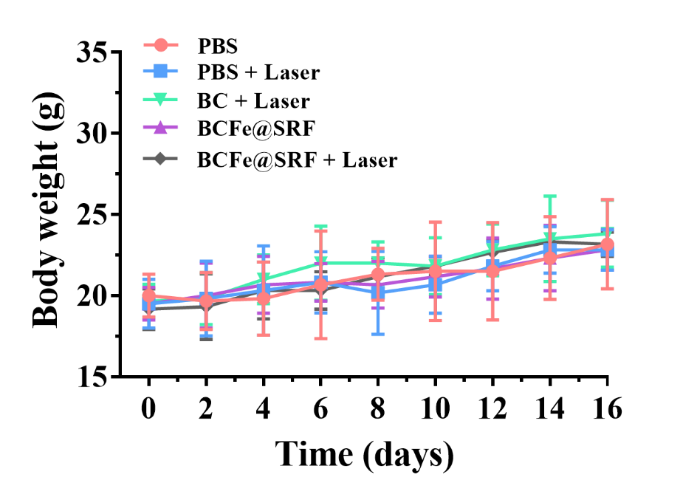


**Figure S15** Mean body weights of the mice (n = 5).


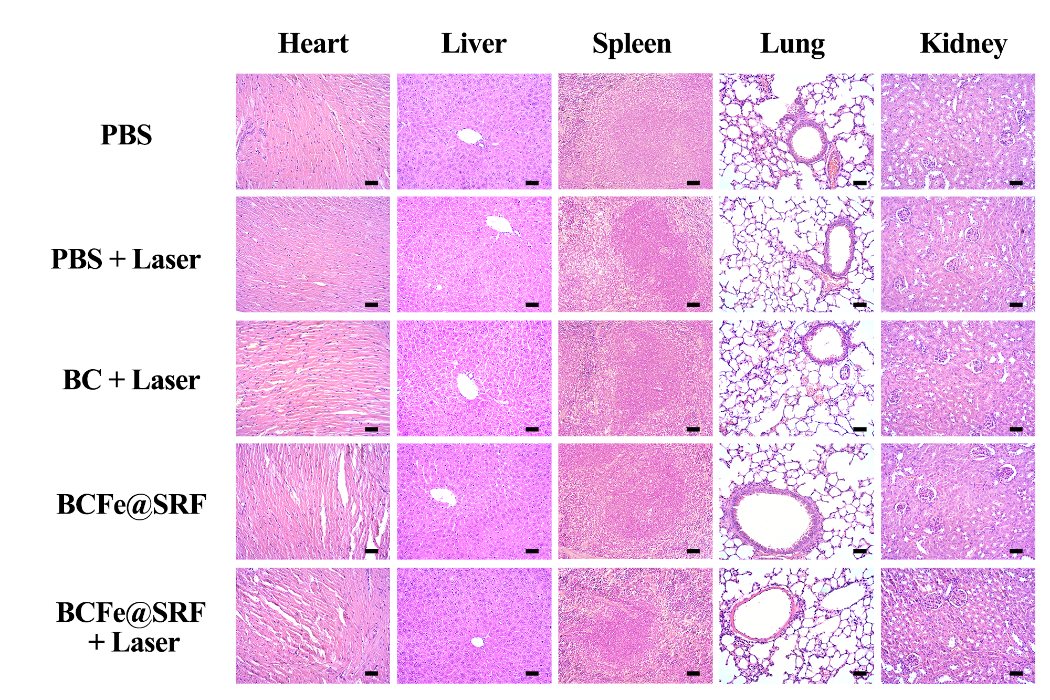


**Figure S16** H&E immuno-histochemical staining of the harvested organs at the end of the indicated treatments. Scale bar: 50 μm.
